# Hyodeoxycholic acid derivatives as liver X receptor α and G-protein-coupled bile acid receptor agonists

**DOI:** 10.1038/srep43290

**Published:** 2017-02-24

**Authors:** Simona De Marino, Adriana Carino, Dario Masullo, Claudia Finamore, Silvia Marchianò, Sabrina Cipriani, Francesco Saverio Di Leva, Bruno Catalanotti, Ettore Novellino, Vittorio Limongelli, Stefano Fiorucci, Angela Zampella

**Affiliations:** 1 Department of Pharmacy, University of Naples “Federico II”, Via D. Montesano, 49, I-80131 Naples, Italy; 2 Department of Surgery and Biomedical Sciences, Nuova Facoltà di Medicina, P.zza L. Severi 1-06132, Perugia, Italy; 3 Università della Svizzera Italiana (USI), Faculty of Informatics, Institute of Computational Science - Center for Computational Medicine in Cardiology, Via G. Buffi 13, CH-6900 Lugano, Switzerland

## Abstract

Bile acids are extensively investigated for their potential in the treatment of human disorders. The liver X receptors (LXRs), activated by oxysterols and by a secondary bile acid named hyodeoxycholic acid (HDCA), have been found essential in the regulation of lipid homeostasis in mammals. Unfortunately, LXRα activates lipogenic enzymes causing accumulation of lipid in the liver. In addition to LXRs, HDCA has been also shown to function as ligand for GPBAR1, a G protein coupled receptor for secondary bile acids whose activation represents a promising approach to liver steatosis. In the present study, we report a library of HDCA derivatives endowed with modulatory activity on the two receptors. The lead optimization of HDCA moiety was rationally driven by the structural information on the binding site of the two targets and results from pharmacological characterization allowed the identification of hyodeoxycholane derivatives with selective agonistic activity toward LXRα and GPBAR1 and notably to the identification of the first example of potent dual LXRα/GPBAR1 agonists. The new chemical entities might hold utility in the treatment of dyslipidemic disorders.

Liver X receptor α and β (LXRs) are ligand activated transcription factors. LXRs function as heterodimers with the retinoid X receptor (RXR) and are activated by naturally occurring cholesterol metabolites known as oxysterols^1^,^2^. LXRα and LXRβ share a high structural homology^3^, but are differentially expressed in mammalian tissues. Thus, while LXRα is primarily expressed in liver, intestine, adipose tissue, and macrophages, LXRβ is ubiquitously expressed.

Upon ligand-induced activation, both LXR isoforms regulate gene expression through binding to LXR response elements (LXREs) in the promoter regions of the target genes. In the liver, LXRα directly induces cytochrome 7A1 (CYP7A1), promoting the conversion of cholesterol into bile acids. In macrophages and adipocytes, LXRs induce the expression of ATP-binding cassettes ABCA1, ABCG1 and apolipoprotein (apoE), increasing the efflux of cholesterol from cells^4^,^5^, and exerts anti-inflammatory activities^6^,^7^ with beneficial effects in rodent models of diabetes and insulin resistance^8^,^9^.

These genetic and pharmacological approaches have shown that LXRs are potentially druggable receptors that might hold utility in the treatment of highly prevalent human diseases including obesity, diabetes, neurodegenerative diseases and chronic inflammatory states^10^,^11^. Unfortunately, the available synthetic agonists for LXRα cause the activation of hepatic lipogenic enzymes, thereby increasing triglyceride synthesis and accumulation, hampering their clinical utility in cardiovascular disease^12^. In mammalians, hyodeoxycholic acid (HDCA, **1** in Fig. 1), a naturally occurring secondary bile acid generated in human small intestine by bacterial C-6 hydroxylation of lithocholic acid (LCA, **2**)^13^, is a weak LXRα agonist^14^. Indeed, HDCA has been shown effective in the treatment of rodent models of metabolic disorders^15^,^16^ and a diet enriched with HDCA was found to protect against atherosclerotic plaques formation in LDL receptor-knockout mice by reducing intestinal cholesterol absorption and increasing HDL-mediated cholesterol efflux from foam cells and macrophages^17^. In addition, HDCA exerts hypolipidemic effect in mice, reducing in liver the gene expression of sterol regulatory element binding protein 1c (SREPB1c), acetyl-CoA carboxylase (ACoA synthase), fatty acid synthase (FAS), and stearoyl-CoA desaturase (S-CoA Des)^18^.

*In vitro* studies have also demonstrated that HDCA is a weak agonist for the G-protein-coupled bile acid receptor GPBAR1 (also known as TGR5), with an EC_50_ of 31.6 μM^19^. GPBAR1 is a membrane bile acid receptor^20^, highly expressed in non-parenchymal liver cells, gallbladder, intestine, heart, spleen, kidney, placenta, leukocytes, skeletal muscle and brown adipose tissue (BAT)^21^. GPBAR1 is preferentially activated by LCA (**2**) and taurolithocholic acid (TLCA, **3** in Fig. 1), with EC_50_ of 0.53 μM and 0.29 μM, respectively^22^.

GPBAR1 activation leads to genomic and non-genomic effects. While non-genomic effects are mediated by the modulation of intracellular concentrations of cAMP, the genomic effects are mediated by the PKA-dependent phosphorylation of CREB (cAMP response element-binding protein), a cellular transcription factor that binds to specific DNA sequences called cAMP response elements (CRE), in the promoter of target genes. In muscles and brown adipose tissue, GPBAR1 increases energy expenditure and oxygen consumption^23^, while in the entero-endocrine L cells, stimulates the secretion of glucagon-like peptide (GLP)-1, an incretin that increases insulin release, thus regulating glucose blood levels, gastrointestinal motility and appetite^24^.

Similarly to LXRs, GPBAR1 is a potentially druggable receptor and might have application in the treatment of metabolic disorders including obesity, diabetes, dyslipidemias, atherosclerosis, liver steatohepatitis, and neurologic disorders^25^,^26^.

In this framework, we have set to explore the chemical space of HDCA with the aim to develop ligands endowed with dual agonist activity towards LXRα and GPBAR1. The newly identified compounds by simultaneously activating LXRα and GPBAR1 could allow targeting metabolic/inflammatory disorders with a novel mechanism of action.

With this background in mind, a large family of hyodeoxycholane derivatives was prepared through various chemical modifications. As shown in Fig. 2, we first introduced on the HDCA scaffold numerous apolar side chains, differing in length, ramification and presence/absence of unsaturation (Subset A). The rationale of this choice relies on the structural features of the ligand-binding site of both LXRα and GPBAR1. In particular, the binding pocket of LXRα is rather amphipathic and thus able to host ligands endowed with both polar and hydrophobic branches^27^. On the other hand, the GPBAR1 ligand-binding pocket presents a small lipophilic cleft that might be targeted by relatively short hydrophobic chains (Fig. 3). Therefore, the introduction on the HDCA steroidal scaffold of hydrophobic side chains with different length can help in deciphering the structural requisites to achieve a dual activity on the two receptors. The obtained set of derivatives was then subjected to a second round of chemical modifications focused on the steroidal scaffold. This step allowed investigating the effect of the A/B ring junction, the stereochemistry at C-3 and the hydroxyl group at C-6 on the ligand affinity towards the receptors (Subset B, Fig. 4). Pharmacological experiments resulted in the identification of several compounds endowed with selective agonistic activity toward LXRα and GPBAR1 and notably to the identification of compound **14**, the first example of potent dual LXRα/GPBAR1 modulator. *In vivo* administration of this modulator, allowed us to investigate the effects of dual LXRα/GPBAR1 activation on mice metabolism.

## Results

### Preparation of Subset A derivatives

Aldehyde **34** was used as a cornerstone intermediate in the preparation of compounds **4**–**12**. A four-step reaction sequence on HDCA, including preparation of methyl ester **31**, protection of alcoholic functions at C-3 and C-6, reduction of the side chain methyl ester and subsequent Swern oxidation furnished aldehyde **34** in quantitative yield (Fig. 5).

As depicted in Fig. 6, Wittig olefination with isopropyl triphenylphosphonium iodide followed by the removal of 3α, 6α-dihydroxy protective groups gave **4** that was also used as starting material in double bound hydrogenation affording the saturated derivative **5** in quantitative yield.

Compounds **6–12**^28^ (Figs 6 and 7) were prepared following the same synthetic protocol and using isobutyl triphenylphosponium iodide, methyl triphenylphosphonium iodide and benzyl triphenylphosphonium iodide, respectively in Wittig olefination.

Figures 8 and 9 illustrate the synthetic protocols for the preparation of HDCA derivatives with *nor* and bis*nor* alkenyl and alkyl side chains. As previously reported^29^, HDCA was subjected to one-carbon degradation at C-24 through the so-called second order “Beckmann rearrangement” affording the 24-*nor*methyl ester **37** (Fig. 8). Protection at C-3 and C-6 hydroxyl groups, followed by reduction of side chain methyl ester and subsequent Swern oxidation furnished key intermediate aldehyde **38** in 94% yield.

Wittig olefination and double bond hydrogenation, in the same operative conditions described for the preparation of derivatives **4**/**5** and **8**/**9**, gave the corresponding *nor* derivatives **13**/**14** and **15**/**16**.

The preparation of C23-analogues **17** and **18** began with the acetylation of HDCA (Fig. 9). Radical oxidative decarboxylation of protected carboxylic acid **39** by treatment with Cu(OAc)_2_ and Pb(OAc)_4_ furnished the ∆^22^ derivative **40**. Sodium methoxide treatment gave the alkene **17** in 90% yield that in turn was also used as starting material to obtain the corresponding saturated derivative **18**.

### Preparation of subset B derivatives

At this point, our chemical speculation was extended to the tetracyclic nucleus exploring the influence of the hydroxyl group at C-6 as well as the configuration of the hydroxyl group at C-3 and the A/B ring junction. Thus, to obtain the corresponding 6-deoxy derivatives **19**–**24**, LCA was subjected to the four-step sequence depicted in Fig. 10 including TBS-protection at C-3, methyl ester formation at C-24, reduction to the corresponding primary alcohol and finally Swern oxidation to obtain aldehyde **41**. Witting olefination with methyl triphenylphosponium iodide and with isopropyl triphenylphosphonium iodide furnished the installation of a terminal alkene and a dimethyl branched alkene as side chain end group in **19** and **21**, respectively. Hydrogenation with Pd(OH)_2_ as catalyst on a small portion of each compound gave the corresponding saturated derivatives **20** and **22**. Finally, oxidative decarboxylation on 3-*O-*acethyl LCA **42** followed by removal of the protecting group at C-3 position gave the alkene **23** that in turn was hydrogenated to the corresponding C23-alkyl derivative **24** (Fig. 10).

In the preparation of 3β-hydroxy-5α-cholane derivatives **25**–**30**, HDCA was transformed in the methyl 3β-hydroxy-5α-cholan-24-oate **43** following our previously published procedure (Fig. 11)^30^.

Then, conversion to aldehyde **44** and Wittig olefination/reduction gave compounds **25**–**28**, following the same synthetic protocol described in Fig. 6.

Intermediate **43** was also used as starting material in the oxidative decarboxylation affording alkene **29** in 62% yield. Hydrogenation at the side chain double bond furnished compound **30**.

### *In vitro* pharmacological evaluation

Derivatives **4–30** were tested for their activity in a luciferase reporter assay with HepG2 and HEK-293T cells transfected with LXRα,β and GPBAR1, respectively. Table 1 reports the efficacy of tested compounds compared to those of reference compounds, GW3965 for LXRα/β and TLCA for GPBAR1.

Each compound was tested at the concentration of 10 μM and transactivation activity of GW3965 on LXRs and TLCA on CRE (i.e. TGR5/GPBAR1) was considered equal to 100%.

As shown in Table 1, the introduction of a hydrophobic side chain on the hyodeoxycholane scaffold (Subset A, compounds **4**–**18**) produced beneficial effects on LXRα. Inspection of biological activity clearly indicates that in the above subset, the efficacy in transactivating LXRα is in correlation with the size of the installed side chain and with the presence of a double bond. The correlation activity/side chain length is not linear with a reduction in LXRα activity for derivatives with too long (compounds **6** and **7**) or too short side chain (compounds **15**–**18**) whereas, as general trend, the presence of a double bond leads to a reduction of the efficacy. Therefore, the best match has been found for compounds **5**, **12** and **14** with an efficacy of 73%, 63% and 109%, respectively.

On the other hand, the length of side chain produces opposite effects on GPBAR1 with improved efficacy of hyodeoxycholane derivatives with shortened side chains (derivatives **13**–**17**).

Analysis of biological data for subset B compounds reveals that the elimination of the hydroxyl group at C-6 is detrimental in term of LXRα transactivation whereas produce positive effects on GPBAR1. Derivatives **19**–**30** shows GPBAR1 efficacy in a 51–96% range.

While the above activity is slightly affected by the configuration of the hydroxyl group at C-3 and of the A/B ring junction, the GPBAR1 efficacy is favored with the shortening of the side chain with compounds **24** and **29**, the most efficacious GPBAR1 selective agonists identified in this study. Of interest the presence of a double bond on the side chain increases the efficacy of the derivatives with *trans* A/B ring junction (compare efficacy of **25** vs **26**, **27** vs **28** and **29** vs **30**).

The behavior of compounds with 5β-configuration is quite the contrary, thus indicating that the introduction of a saturated side chain produces beneficial effects on bent shaped nuclei (compare efficacy of **24** vs **23**). None of tested compounds was able to transactivate LXRβ (Table 1) and FXR (Figure S1).

Table 2 shows EC_50_ values of the most efficacious compounds identified in this study.

Compounds **14** was further investigated *in vitro* to evaluate its effects on LXRα and GPBAR1 target genes by RT-PCR. The HepG2 and Glutag cells (1 × 10^6^) were plated and, after 24 hours of starvation, were stimulated with receptor agonists GW3965, TLCA and HDCA (10 μM) and with increasing concentration of compound **14** (1, 5, 10, 25, 50 μM). As shown in Fig. 12, compound **14** was able to induce the expression of ABCA1 and SREBP1c genes in HepG2 cells in dose-dependent manner with an EC_50_ of 8.3 μM and 5.8 μM respectively.

The compound was also able to activate the expression of pro-glucagon mRNA in Glutag cells; however, the induction is dose-dependent only until the 10 μM concentration with an EC_50_ of 6.5 μM. These results demonstrate that this compound is a potent, effective and selective LXRα and GPBAR1 dual agonist.

Compound **14** was also investigated *in vivo* to verify whether the LXRα activation causes lipid accumulation in the liver. C57BL6 mice were administered with **14** (30 mg/Kg daily by oral gavage) for two weeks. As showed in Fig. 13, no effects were observed in mice treated with compound **14** on the plasmatic levels of AST, cholesterol and triglycerides (Fig. 13A). Liver histology (H&E staining), in which no differences were observed between control group and mice treated with **14** (Fig. 13B), confirmed this result. Real-Time PCR assayed on liver tissue demonstrated that the compound does not induce the expression of steatosis markers genes, FAS, SREBP1c, CD36 and PPARs (Fig. 13C). Of interest, compound **14** increases the expression of GPBAR1 target genes GLP1 and Fgf21 in terminal ileum (Fig. 13D). These results demonstrate that, despite its activity on LXRα, compound **14** does not induce lipid accumulation and liver steatosis and this positive effect is closely related to the simultaneous activation of GPBAR1, as evidenced by the *in vivo* induction of GPBAR1 target genes in the gut.

### Molecular Modeling

In order to investigate the molecular bases of the dual LXRα/GPBAR1 activity of **14**, a thorough computational study has been carried out. First, we performed docking calculations of **14** in the homology model of GPBAR1 that we have previously developed and successfully used for drug design^31^. The best scored docking pose (Fig. 14A) shows that **14** binds to GPBAR1 similarly to other bile acids recently reported by us as agonists of this receptor^31^,^32^,^33^. Nevertheless, some differences can be found. In detail, while the ligand 3α-hydroxyl group engages the typical H-bond interaction with the Glu169 side chain, the hydrophobic side chain of **14** occupies the small lipophilic pocket formed by Ala66, Leu68 and Leu71 on TM2. This orientation of the side chain in the binding site is different respect to that of the derivatives with polar functional groups on the side chain, which occupy the site interacting with the serine residues of transmembrane helices TM7 and TM1. The ligand binding mode is further stabilized by a set of hydrophobic interactions established by the steroidal scaffold with the side chains of Leu71, Phe96, Leu174 and Trp237.

In order to elucidate the binding mode of **14** to LXRα, docking simulations were performed using the crystal structure of the ligand binding domain (LBD) of the receptor (PDB code: 3IPU)^34^. In this case, docking calculations suggest two possible binding modes, A and B, where the ligand assumes two opposite orientations in the LBD (Figure S2). Specifically, in A the hydrophobic chain of **14** is oriented towards the helices 11 and 12 of LXRα, while the 3α- and 6α-hydroxyl groups interact with the residues of the β-sheet close to H1. In B, the steroidal scaffold is oriented in the opposite direction relative to A in the LBD. In particular, the 3α- and 6α-hydroxyl groups are close to His421 of helix 11, while the hydrophobic side chain extends towards the β-sheet in the ligand binding pocket. We decided to further investigate the two binding modes assessing their stability through over 100 ns molecular dynamics (MD) calculations. In particular, we evaluated, during the simulation, the conservation of the interactions engaged by the ligand with the protein and the geometrical stability of the ligand by computing the root mean square displacement (rmsd) of its heavy atoms relative to their starting position (see Figure S3 for details). In B, the original ligand/protein interactions, such as the H-bond between **14** and His421, are lost and the ligand rmsd value increases during the simulation showing instability of this binding mode. At variance with B, in A the ligand rmsd values are low (Figure S3) and all the starting ligand/protein interactions are conserved throughout the simulation. The MD results prompted us to consider only binding mode A for further analysis. In this pose (Fig. 14B and S4), the steroidal scaffold of **14** establishes favorable contacts with lipophilic residues such as Phe257, Phe315 and Phe326, while the 3α-group of **14** H-bonds with the Ser264 side chain. Additional water-mediated interactions are engaged by both the ligand hydroxyl groups with the side chains of Arg232, Glu267 and Glu301. On the other hand, the ligand hydrophobic side chain inserts into a deep lipophilic pocket shaped by helices (H) 3, 4, 6, 11, and 12, where it can establish favorable contacts with several residues such as Phe254, Phe335, Leu435, Leu439 and Trp443. This hydrophobic network stabilizes the position of Trp443 in the binding site favoring its interaction through a cation-π interaction with His421. Such interaction is considered fundamental for the activation of nuclear receptors like LXRα, since it allows the C-terminal H12 to adopt a conformation competent for the binding of co-activator peptides. This event triggers in turn the LXRα/RXR dimerization and the transcription of target genes^27^. Although **14** is devoid of a polar side chain, its binding mode to LXRα is overall similar to that reported for some oxysterols^27^ and to the crystallographic binding pose of 24(*S*), 25-epoxycholesterol in LXRβ (Figure S4)^35^. Furthermore, the binding mode of **14** is in line with the mutagenesis data that suggest a functional role of Glu267 in the binding of oxysterols to LXRs^27^.

## Discussion and Conclusion

**Figure 2:**
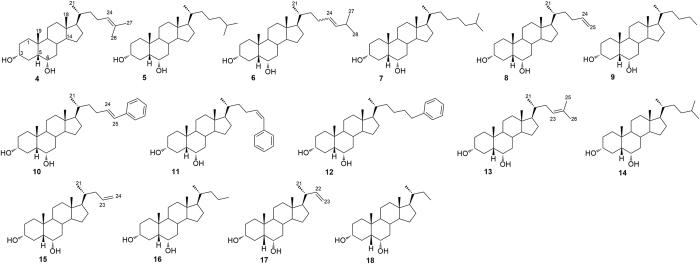
Subset A: installation of a hydrophobic side chain on hyodeoxycholane scaffold.

**Figure 4:**
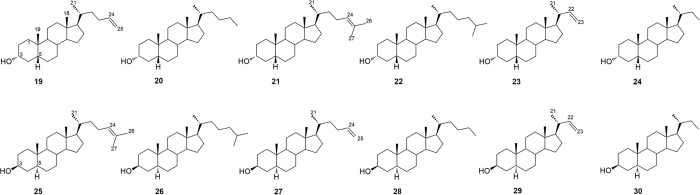
Subset B: installation of a hydrophobic side chain on modified A-B ring hyodeoxycholane scaffold.

**Figure 5:**
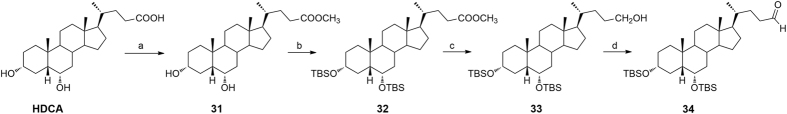
Synthesis of key aldehyde 34. *Reagents and conditions*: (**a**) p-TsOH, MeOH dry, quantitative yield; (**b**) 2,6-lutidine, t-butyldimethylsilyltrifluoromethanesulfonate, CH_2_Cl_2_, 0 °C, quantitative yield; (**c**) LiBH_4_, MeOH dry, THF, 0 °C, 56%; (**d**) DMSO, oxalyl chloride, TEA dry, CH_2_Cl_2_, −78 °C, quantitative yield.

**Figure 6:**
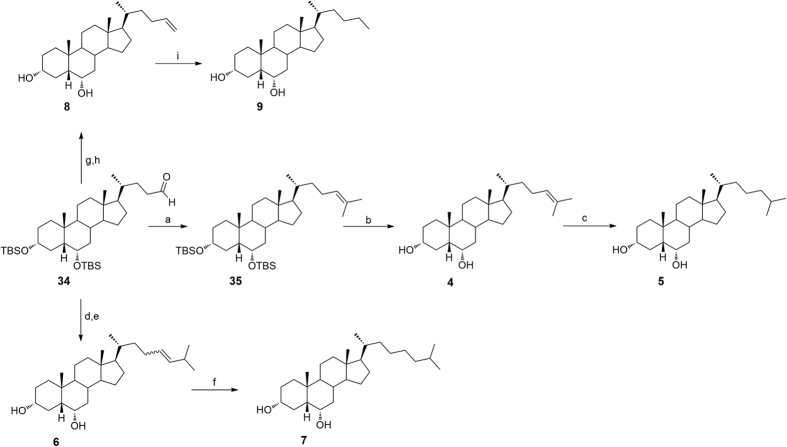
Preparation of Subset A derivatives: introduction of C25 linear and C26/C27 branched aliphatic side chains on hyodeoxycholane scaffold. *Reagents and conditions*: (**a**) *n*-BuLi, isopropyl triphenylphosponium iodide, THF dry, r.t., 84%; (**b**) HCl 37%, MeOH, quantitative yield; (**c**) H_2_, Pd(OH)_2_ degussa type, THF:MeOH dry 1:1, quantitative yield; (**d**) *n*-BuLi, isobutyl triphenylphosponium iodide, THF dry, r.t.; (**e**) HCl 37%, MeOH; (**f**) H_2_, Pd(OH)_2_ degussa type, THF:MeOH dry 1:1; (**g**) *n*-BuLi, methyl triphenylphosponium iodide, THF dry, r.t, 60%; (**h**) HCl 37%, MeOH, quantitative yield; (**i**) H_2_, Pd(OH)_2_ degussa type, THF:MeOH dry 1:1, 70%.

**Figure 7:**
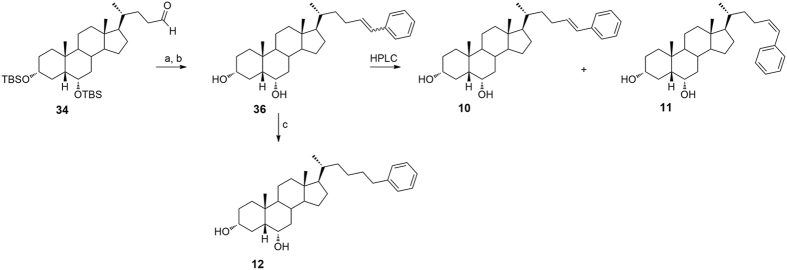
Preparation of Subset A derivatives: introduction of an aromatic end-group side chain on hyodeoxycholane scaffold. *Reagents and conditions*: (**a**) *n*-BuLi, benzyl triphenylphosponiumiodide, THF dry; (**b**) HCl 37%, MeOH, 67% over two steps; (**c**) H_2_, Pd(OH)_2_ degussa type, THF:MeOH dry 1:1, quantitative yield.

**Figure 8:**
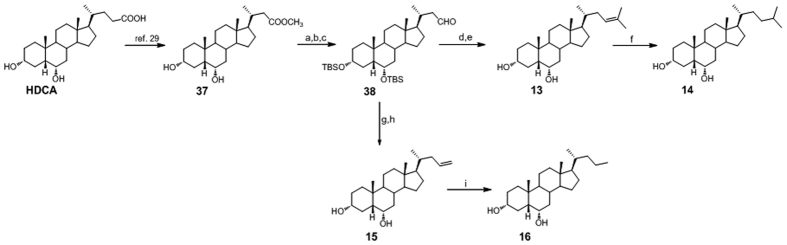
Preparation of Subset A derivatives: introduction of C24 linear and C25 branched aliphatic side chains on hyodeoxycholane scaffold. *Reagents and conditions*: (**a**) 2,6-lutidine, t-butyldimethylsilyltrifluoromethanesulfonate, CH_2_Cl_2_, 0 °C; (**b**) LiBH_4_, MeOH dry in THF dry; (**c**) DMSO, oxalyl chloride, TEA dry, CH_2_Cl_2_, −78 °C, 94% over three steps; (**d**) *n*-BuLi, isopropyl triphenylphosphonium iodide, THF dry, r.t.; (**e**) HCl 37%, MeOH, 80% over two steps; (**f**) H_2_, Pd(OH)_2_ degussa type, THF/MeOH 1:1, 90%; (**g**) *n*-BuLi, methyl triphenylphosphonium iodide, THF dry; (**h**) HCl 37%, MeOH, 95% over two steps; (**i**) H_2_, Pd(OH)_2_ degussa type, THF/MeOH dry 1:1, 88%.

**Figure 9:**
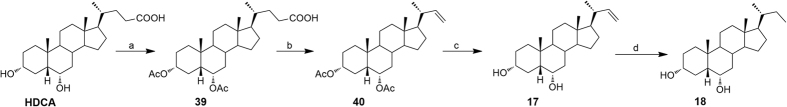
Preparation of Subset A derivatives: introduction of a C23 linear aliphatic side chain on hyodeoxycholane scaffold. *Reagents and conditions*: (**a**) Ac_2_O, pyridine, quantitative yield; (**b**) Cu(OAc)_2_ H_2_O, Pb(OAc)_4_ in toluene dry/pyridine dry, 17%; (**c**) CH_3_ONa, CHCl_3_ dry/MeOH dry 5:3 v/v, 90%; (**d**) H_2_, Pd(OH)_2_ degussa type, THF dry/MeOH dry 1:1 v/v, quantitative yield.

**Figure 10:**
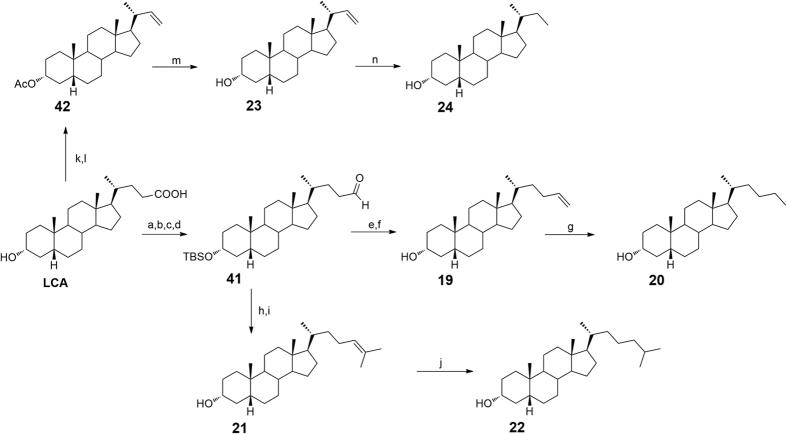
Preparation of Subset B derivatives. Linear C23/C25 and branched C26 aliphatic side chains on 6-deoxyhyodeoxycholane scaffold. *Reagents and conditions*: (**a**) *p*-TsOH, MeOH dry; (**b**) 2,6-lutidine, t-butyldimethylsilyltrifluoromethanesulfonate, CH_2_Cl_2_, 0 °C; (**c**) LiBH_4_, MeOH dry, THF, 0 °C; (**d**) DMSO, oxalyl chloride, TEA dry, CH_2_Cl_2_, −78 °C, 72% over four steps; (**e**) *n*-BuLi, methyl triphenylphosponium iodide, THF dry, r.t.; (**f**) HCl 37%, MeOH, quantitative yield over two steps; (**g**) H_2_, Pd(OH)_2_ degussa type, THF:MeOH dry 1:1, 86%; (**h**) *n*-BuLi, isopropyl triphenylphosponium iodide, THF dry, r.t.; (**i**) HCl 37%, MeOH, 40% over two steps; (**j**) H_2_, Pd(OH)_2_ degussa type, THF:MeOH dry 1:1, quantitative yield; (**k**) Ac_2_O, Pyr; (**l**) Cu(OAc)_2_ H_2_O, Pb(OAc)_4_ in toluene dry/pyridine dry, quantitative yield over two steps; (**m**) CH_3_ONa, CHCl_3_ dry/MeOH dry 5:3 v/v, 27%; (**n**) H_2_, Pd(OH)_2_ degussa type, THF dry/MeOH dry 1:1 v/v, 22%.

**Figure 11:**
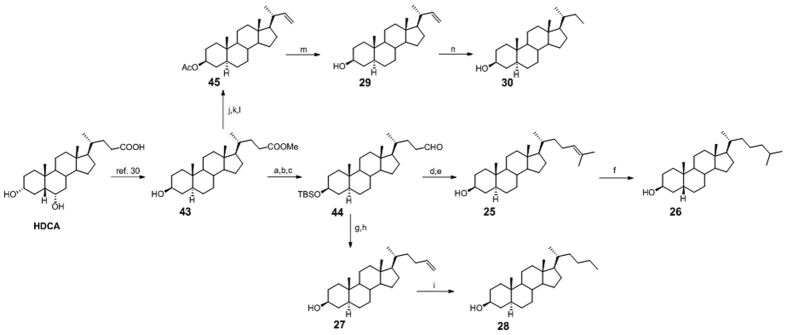
Preparation of Subset B derivatives. Linear C23/C25 and branched C26 aliphatic side chains on 3β-hydroxy-6-deoxy-5α-hyodeoxycholane scaffold. *Reagents and conditions*: (**a**) 2,6-lutidine, t-butyldimethylsilyltrifluoromethanesulfonate, CH_2_Cl_2_, 0 °C; (**b**) LiBH_4_, MeOH dry, THF, 0 °C; (**c**) DMSO, oxalyl chloride, TEA dry, CH_2_Cl_2_, −78 °C, 34% over three steps; (**d**) *n*-BuLi, isopropyl triphenylphosponium iodide, THF dry, r.t.; (**e**) HCl 37%, MeOH, 34% over two steps; (**f**) H_2_, Pd(OH)_2_ degussa type, THF:MeOH dry 1:1, quantitative yield; (**g**) *n*-BuLi, methyl triphenylphosponium iodide, THF dry, r.t.; (**h**) HCl 37%, MeOH, 38% over two steps; (**i**) H_2_, Pd(OH)_2_ degussa type, THF:MeOH dry 1:1, quantitative yield; (**j**) NaOH, MeOH/H_2_O 1:1 v/v, reflux; (**k**) Ac_2_O, pyridine; (**l**) Cu(OAc)_2_ H_2_O, Pb(OAc)_4_ in toluene dry/pyridine dry, 78%; (**m**) CH_3_ONa, CHCl_3_ dry/MeOH dry 5:3 v/v, 62%; (**n**) H_2_, Pd(OH)_2_ degussa type, THF dry/MeOH dry 1:1 v/v, quantitative yield.

**Figure 12:**
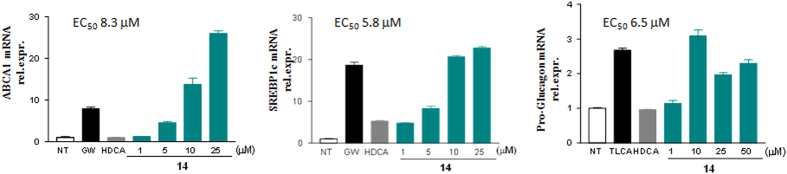
Quantitative Real-Time PCR analysis of mRNA expression on LXRα and GPBAR1 target genes. ABCA1 (**A**) and SREBP1c (**B**) expression in HepG2 cells primed with increasing concentration of compound **14** (1, 5, 10 and 25 μM). GW3965 and HDCA were used as positive controls. Pro-glucagon (**C**) expression in Glutag cells stimulated with increasing dose of compound **14** (1, 10, 25 and 50 μM). TLCA and HDCA were used as a positive control. Values are normalized to GAPDH and are expressed relative to those of not treated cells (NT) which are arbitrarily settled to 1. The relative mRNA expression is expressed as 2^(−ΔΔCt)^.

**Figure 13:**
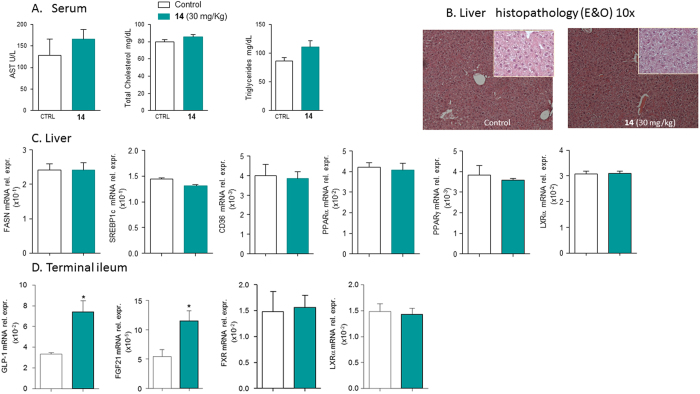
Effects of compound 14 on hepatic lipid metabolism and on terminal ileum after administration on intact mice. C57BL6 mice were treated with **14** (30 mg/Kg daily per os) for two weeks. Results are the mean ± SE of 3–5 mice per group; *p < 0.05 versus control mice. (**A**) Serum levels of AST, total cholesterol and triglycerides; (**B**) Histopathology analysis (Hematoxilin and Eosin) of liver sections. Magnification 4x. Insets magnification 40x. The images show that no fat deposition occurs in the liver of mice treated up to 2 weeks with **14** at the dose of 30 mg/Kg; (**C**) Relative hepatic mRNA expression of genes involved in fatty acids metabolism (FASN, SREBP1C, CD36) and genes for nuclear receptors (PPARα, PPARγ, LXR α); (**D**) Relative hepatic mRNA expression of GLP-1, FGF21, FXR and LXRα genes in terminal ileum.

**Figure 14:**
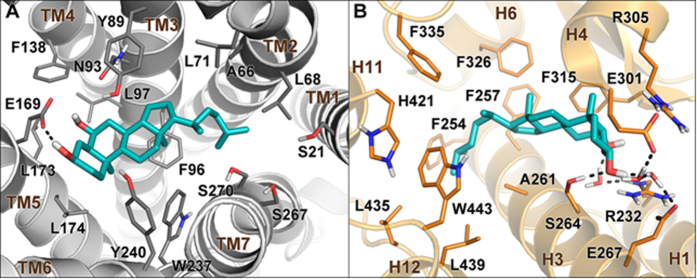
Binding mode of 14 in GPBAR1 and in LXRα. (**A**) Docking pose in the homology model of GPBAR1. (**B**) Conformation obtained from MD simulations within the LXRα LBD (PDB code: 3IPU). Compound **14** is represented as cyan sticks. GPBAR1 and LXRα are shown as gray and orange cartoons, respectively. Amino acids important for ligand binding are depicted as sticks. Non-polar hydrogens are omitted for clarity.

Understanding the structural requisites for selective affinity of a ligand towards its molecular target is of paramount relevance in drug design. This task can be particularly difficult when proteins involved in different cellular pathways discriminate among ligands with small chemical changes as in the case of bile acid receptors. On the other hand, one might exploit the possibility to simultaneously activate more than one molecular target developing multi-target compounds that could be beneficial from the therapeutic point of view. In the present work, we have explored the chemical space of hyodeoxycholic acid (HDCA) introducing a hydrophobic side chain on hyodeoxycholane scaffold and on A/B ring modified hyodeoxycholane scaffold. Several selective LXRα and GPBAR1 agonists as well the first example of LXRα/GPBAR1 dual modulators have been identified. The lead optimization of HDCA was rationally driven by the structural informations on the binding site of the two targets. Moreover, the information coming from binding calculations of **14** in GPBAR1 and LXRα and the pharmacological activity of structurally related compounds, allowed us to delineate the structural requirements for ligand binding to either receptor. In particular, the binding mode of **14** to LXRα suggests that the 3α- and 6α-hydroxyl groups as well as C25/C26 hydrophobic chains are crucial for ligand activity. In fact, compounds without the 6α-OH group (**19**–**30**) or endowed with too short side chains (linear C24/C23, compounds **15–18**) and too long (branched C27, compounds **6** and **7**) are inactive towards LXRα. At variance with LXRα, the 6α-hydroxyl group is not a prerequisite for ligand binding to GPBAR1, with compounds **19**–**30** invariably able in transactivating GPBAR1 (Table 1), while in this receptor, compounds with a hydrophobic side chain longer than C25 are inactive since their lipophilic branch can difficulty insert into the small hydrophobic cleft of the receptor binding site. Overall, our results lead to conclude that subset A derivatives (Fig. 2) with linear C25 (compounds **8** and **9**), phenyl-substituted C25 (compound **12**) and methyl-branched C26 (compounds **4** and **5**) side chains are LXRα selective. On the other hand, subset A derivatives with linear C24/C23 side chains (compounds **15**–**17**) and compounds without the 6α-hydroxyl group (Subset B in Fig. 4) turn out to be active only on GPBAR1. Finally, the concurrent presence of the 6α-OH group and of a C-25 methyl-branched side chain as in compounds **13** and **14** allow achieving dual LXRα/GPBAR1 activity.

Compound **14** was effective in modulating the expression of canonical LXRα and GPBAR1 target genes. We have shown that **14** increases the expression of SREPB1c, ABCG1 and GLP1 *in vitro*. Noteworthy, *in vivo* administration of compound **14** on intact mice demonstrated beneficial effects. Compound **14** does not induced the typical effects of LXRα agonists, which usually activate lipogenic enzymes causing accumulation of lipid in the liver. Conversely, the expression of steatosis marker genes FAS, SREBP1c and CD36 was not modulated compared to the control group. This positive effect is closely related to the simultaneous activation of GPBAR1, as demonstrated by the *in vivo* induction of GLP1 expression in the gut. Collectively, these data strongly support a further pharmacological characterization of the newly discovered agent in rodent models of metabolic disorders. In summary, we have generated a novel series of HDCA derivatives that allowed targeting metabolic disorders including diabetes, chronic inflammatory states and neurodegenerative diseases, by exploiting a completely novel mechanism of action, i.e. the simultaneous activation of LXRα and GPBAR1.

## Methods

### Chemistry

High-resolution ESI-MS spectra were performed with a Micromass Q-TOF mass spectrometer. NMR spectra were obtained on Varian Inova 400 NMR spectrometer (^1^H at 400, MHz, ^13^C at 100 MHz, respectively) equipped with a SUN microsystem ultra 5 hardware and recorded in CD_3_OD (δ_H_ = 3.31 and δ_C_ = 49.0 ppm) and CDCl_3_ (δ_H_ = 7.26 and δ_C_ = 77.0 ppm). All of the detected signals were in accordance with the proposed structures. Coupling constants (*J* values) are given in Hertz (Hz), and chemical shifts (δ) are reported in ppm and referred to CHD_2_OD and CHCl_3_ as internal standards. Spin multiplicities are given as s (singlet), br s (broad singlet), d (doublet), or m (multiplet).

HPLC was performed with a Waters Model 510 pump equipped with Waters Rheodine injector and a differential refractometer, model 401. Reaction progress was monitored via thin-layer chromatography (TLC) on Alugram silica gel G/UV254 plates. Silica gel MN Kieselgel 60 (70–230 mesh) from Macherey-Nagel Company was used for column chromatography. All chemicals were obtained from Sigma-Aldrich, Inc. Solvents and reagents were used as supplied from commercial sources with the following exceptions. Dichloromethane, tetrahydrofuran and trimethylamine were distilled from calcium hydride immediately prior to use. Methanol was dried from magnesium methoxide as follow. Magnesium turnings (5 g) and iodine (0.5 g) were refluxed in a small (50–100 mL) quantity of methanol until all of the magnesium has reacted. The mixture was diluted (up to 1 L) with reagent grade methanol, refluxed for 2–3 h then distilled under nitrogen. All reactions were carried out under argon atmosphere using flame-dried glassware.

The purities of compounds were determined to be greater than 95% by HPLC.

### Synthetic procedures

See the Supporting Information.

### Cell culture

HepG2, an immortalized human epatocarcinoma cell line, was cultured and maintained at 37 °C and 5% CO_2_ in E-MEM additioned with 10% FBS, 1% glutamine and 1% penicillin/streptomycin. HEK-293T and Glutag cells were cultured and maintained at 37 °C and 5% CO_2_ in D-MEM additioned with 10% FBS, 1% glutamine and 1% penicillin/streptomycin.

### Luciferase reporter gene assay and dose-response curves

To evaluate LXRα mediated transactivation, HepG2 cells were transfected with 20 ng of the reporter vector p(UAS)5XTKLuc, 100 ng of a vector containing the ligand binding domain of LXRα cloned upstream of the GAL4-DNA binding domain (i.e. pSG5-LXRαLBD-GAL4DBD) and 100 of pGL4.70 (Promega), a vector encoding the human Renilla gene. To evaluate GPBAR1 mediated transactivation, HEK-293T cells were transfected with 200 ng of human pGL4.29 (Promega), a reporter vector containing a cAMP response element (CRE) that drives the transcription of the luciferase reporter gene luc2P, with 100 ng of pCMVSPORT6-human GPBAR1, and with 100 ng of pGL4.70 Renilla. To evaluate FXR mediated transactivation, HepG2 cells were transfected with 100 ng of human pSG5-FXR, 100 ng of human pSG5-RXR, 200 ng of the reporter vector p(hsp27)-TK-LUC containing the FXR response element IR1 cloned from the promoter of heat shock protein 27 (hsp27) and with 100 ng of pGL4.70 Renilla. At 24 h post-transfection, cells were stimulated 18 h with 10 μM GW3965, TLCA, or CDCA and compounds **4**–**30** (10 μM). Luciferase activities were assayed and normalized with Renilla activities. Dose-response curves were performed in HepG2 and HEK-293T cells transfected as described above and then treated with increasing concentrations of compounds **4**, **5**, **8**, **9**, **12**–**15**, **17**, **20**, **21**, **24**, **25**, **27**, **29**, **30** (1, 5, 10, 25 and 50 μM). At 18 h post stimulations, cellular lysates were assayed for luciferase and Renilla activities using the Dual-Luciferase Reporter assay system (E1980, Promega). Luminescence was measured using Glomax 20/20 luminometer (Promega). Luciferase activities (RLU) were normalized with Renilla activities (RRU).

### Animal model

C57BL6 mice were originally donated by Dr. Galya Vassileva (Schering-Plough Research Institute, Kenilworth). The colonies were maintained in the animal facility of University of Perugia. Mice were treated with compound **14** (30 mg/Kg daily by oral gavage) or vehicle (distilled water) for two weeks. Mice were housed under controlled temperatures (22 °C) and photoperiods (12:12-h light/dark cycle), allowed unrestricted access to standard mouse chow and tap water and allowed to acclimate to these conditions for at least 5 days before inclusion in an experiment. The study was conducted in agreement with the Italian law and the protocol was approved by ethical committee of University of Perugia and by National committee of Ministry of Health (permission n. 42/2014-B). The Veterinarian monitored the health and body conditions of the animals daily in the animal facility. The study protocol caused minor suffering; however, animals that lost more than 25% of the initial body weight were euthanized. At the day of sacrifice mice were deeply anesthetized with a mixture of tiletamine hypochlorite and zolazepam hypochlorite/xylazine at a dose of 50/5 mg/Kg. Blood, liver and terminal ileum were collected for further analysis. Aspartate aminotransferase (AST), total cholesterol and triglycerides were measured by routine biochemical clinical chemistry. For histological examination, portions of liver lobes were fixed in 10% formalin, embedded in paraffin, sectioned (5 μm thin) and stained with Hematoxylin/Eosin (H&E) for morphometric analysis.

### RNA isolation and RT-PCR

HepG2 and Glutag cells were plated at 1 × 10^6 ^cells/well in a 6 well plate. After an overnight incubation, cells were starved and then stimulated for 18 h with GW3965 or TLCA (10 μM), HDCA (10 μM), and with increasing concentrations of compound **14** (1, 5, 10, 25, 50 μM).

Total RNA was isolated from HepG2 or Glutag cells and from animal tissues (liver and terminal ileum) using the TRIzol reagent according to the manufacturer’s specifications (Invitrogen). One microgram of purified RNA was treated with DNase-I and reverse transcribed with Superscript II (Invitrogen). For Real Time PCR, 25 ng template was dissolved in 25 μL containing 200 nmol/L of each primer and 12.5 μL of 2 × SYBR FAST Universal ready mix (Invitrogen). All reactions were performed in triplicate, and the thermal cycling conditions were as follows: 2 min at 95 °C, followed by 40 cycles of 95 °C for 20 s and 60 °C for 30 s in StepOnePlus (Applied Biosystems). The relative mRNA expression was calculated and expressed as 2^(−ΔΔCt)^. Forward and reverse primer sequences were the following: human GAPDH, gaaggtgaaggtcggagt and catgggtggaatcatattggaa; human ABCA1, gcttgggaagatttatgacagg and aggggatgattgaaagcagtaa; human SREBP1c, gcaaggccatcgactacatt and ggtcagtgtgtcctccacct; mouse GAPDH, ctgagtatgtcgtggagtctac and gttggtggtgcaggatgcattg; mouse Pro-glucagon, tgaagacaaacgccactcac and caatgttgttccggttcctc; mouse FAS, tcaagatgaaggtggcagaggtgct and ttgagcagtgccgggattcgg; mouse SREBP1c, gatcaaagaggagccagtgc and tagatggtggctgctgagtg; mouse CD36, cggagacatgcttattgagaa and actctgtatgtgtaaggacct; mouse PPARα, cagaggtccgattcttccac and gatcagcatcccgtgtttgt; mouse PPARγ, gccagtttcgatccgtagaa and aatccttggccctctgagat; mouse LXRα, ggctcaccagcttcattagc and gcaggaccagctccaagtag; mouse FXR, tgtgagggctgcaaaggttt and acatccccatctctctgcac; mouse Fgf21, acacagatgacgaccaagacac and aagtgaggcgatccatagagag.

### Molecular docking

The Glide (version 7.1) software package^36^ was used to perform molecular docking calculations in the three-dimensional model of hGPBAR1^31^ and in the crystal structure of LXRα-LBD bound to a synthetic benzisoxazole urea agonist (PDB code: 3IPU)^34^.

This structure was selected among the several available using the following criteria: i) the higher resolution of the electron density map; ii) the presence of all amino acids in helix 1, which is not fully resolved in all the LXRα crystal structures; iii) the presence of an agonist with bulkiness comparable to bile acid derivatives. Missing residues in the loop connecting H1 with H3 were added and refined using Prime^37^. Ligand and receptors structures were prepared as described in a previous paper^32^.

For both GPBAR1 and LXRα, a box of 30 × 30 × 30 Å centered on the ligand binding cavities was created. The standard precision (SP) mode of the GlideScore function^38^,^39^ was used to score the predicted binding poses.

### Molecular dynamics

All the simulations were performed with NAMD 2.10^40^ using the *ff14SB*^41^ and *gaff*^42^ Amber force field parameters for the protein and the ligand, respectively. Each complex was solvated in a 12.0 Å layer cubic water box using the TIP3P water model parameters^43^. The addition of 2 Na^+^ ions ensured neutrality. Amber charges were applied to the proteins and water molecules, whereas the ligand charges were computed using the restrained electrostatic potential (RESP) fitting procedure^44^. The ESP was first calculated by means of the Gaussian09 package^45^ using a 6–31 G* basis set at Hartree-Fock level of theory, and then the RESP charges were obtained by a two-stages fitting procedure using Antechamber^46^. A 10 Å cutoff (switched at 8.0 Å) was used for atom-pair interactions^47^,^48^. The long-range electrostatic interactions were computed by means of the particle mesh Ewald (PME) method^49^, using a 1.0 Å grid spacing in periodic boundary conditions. The SHAKE algorithm was applied to constraint bonds involving hydrogen atoms, and thus an integration time step of 2 fs could be used. Each complex was heated up to 300 K while putting harmonic constraints on the protein and the ligand, which were gradually released along the thermalization process. Production runs were then performed under NPT conditions at 1 atm and 300 K.

All figures were rendered using PyMOL (http://www.pymol.org).

## Additional Information

**How to cite this article:** De Marino, S. *et al*. Hyodeoxycholic acid derivatives as liver X receptor α and G-protein-coupled bile acid receptor agonists. *Sci. Rep.* **7**, 43290; doi: 10.1038/srep43290 (2017).

**Publisher's note:** Springer Nature remains neutral with regard to jurisdictional claims in published maps and institutional affiliations.

## Supplementary Material

Supplementary Information

## Figures and Tables

**Figure 1:**
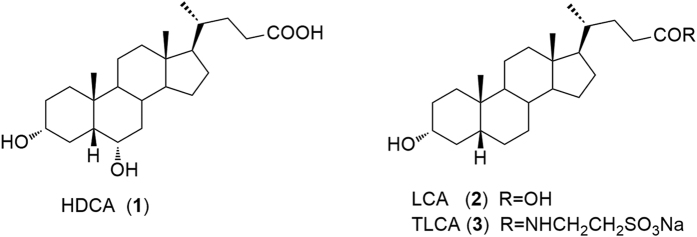
Naturally occurring bile acids. HDCA, a weak LXRα/GPBAR1 dual agonist and LCA and its tauro-conjugated derivative, TLCA, the most potent endogenous activators of GPBAR1.

**Figure 3:**
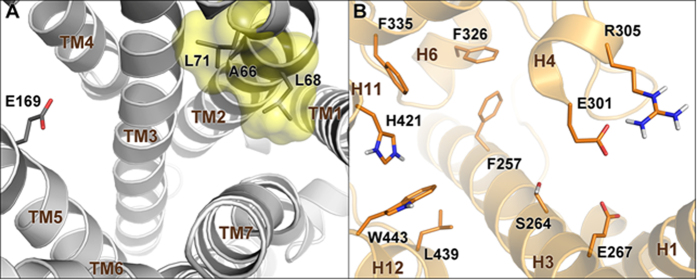
Ligand binding sites of GPBAR1 and LXRα. In (**A**) the amino acids defining the small lipophilic cleft in GPBAR1 are highlighted as yellow transparent surface. (**B**) Shows the amphipathic nature of LXRα-LBD characterized by the presence of both polar and hydrophobic residues. GPBAR1 and LXRα are shown as gray and orange cartoons, respectively. In both receptors, representative residues are depicted as sticks. Non-polar hydrogens are omitted for clarity.

**Table 1:** GPBAR1 and LXRs efficacy of compounds 4–30.

Compound	GPBAR1*		LXRα**		LXRβ
	Efficacy		Efficacy		Efficacy
	(% vs TLCA)	(% vs HDCA)	(% vs GW3965)	(% vs HDCA)	(% vs GW3965)
**HDCA**	26		15, 5		NA***
**4**	26, 3	101, 0	49, 3	317, 1	NA
**5**	14, 9	57, 5	72, 8	468, 1	NA
**6**	65, 0	249, 8	32, 6	209, 7	NA
**7**	26, 3	101, 2	28, 0	180, 1	NA
**8**	25, 5	98, 0	42, 9	276, 3	NA
**9**	27, 0	103, 9	54, 1	348, 3	NA
**10**	26, 0	100, 0	12, 2	78, 5	NA
**11**	33, 2	127, 7	26, 2	168, 3	NA
**12**	18, 6	71, 7	63, 1	406, 1	NA
**13**	52, 6	202, 4	36, 1	232, 0	NA
**14**	55, 1	211, 9	109, 3	703, 3	NA
**15**	68, 7	264, 0	NA	—	NA
**16**	53, 7	206, 6	23, 8	153, 0	NA
**17**	53, 1	204, 3	17, 3	111, 7	NA
**18**	22, 8	87, 6	NA	—	NA
**19**	75, 1	288, 7	NA	—	NA
**20**	74, 0	284, 6	20, 0	128, 6	NA
**21**	73, 0	280, 7	NA	—	NA
**22**	77, 1	296, 5	NA	—	NA
**23**	59, 6	229, 1	NA	—	NA
**24**	92, 9	357, 1	NA	—	NA
**25**	58, 1	223, 2	NA	—	NA
**26**	50, 9	195, 8	NA	—	NA
**27**	64, 4	247, 7	NA	—	NA
**28**	54, 9	211, 1	NA	—	NA
**29**	96, 0	368, 8	15, 0	96, 7	NA
**30**	58, 1	223, 5	NA	—	NA

^*^Activity toward GPBAR1 in a reporter assay was assessed in HEK-293T cells transfected with a cAMP responsive element (CRE) cloned upstream to the luciferase gene. For calculation of efficacy data, maximal transactivation of CRE caused by each compound (10 μM) was compared to maximal transactivation caused by TLCA (10 μM) and by HDCA (10 μM).

^**^Activity toward LXRα in a reporter assay was assessed in HepG2 cells transfected with an LXRα responsive element (LRE) cloned upstream to the luciferase gene. For calculation of efficacy data, maximal transactivation of LRE caused by each compound (10 μM) was compared to maximal transactivation caused by GW3965 (10 μM) and by HDCA (10 μM).

^***^NA: no activity at 10 μM.

**Table 2:** EC_50_ values for selected compounds.

	GPBAR1	LXRα
Compound	Affinity (μM)*	Affinity (μM)
Selective LXRα agonists		
4		6.99 ± 0.31
5		8.2 ± 0.16
8		2.7 ± 0.65
9		5.1 ± 0.43
12		12.4 ± 0.41
GPBAR1/LXRα dual agonists		
13	4.2 ± 0.79	22.3 ± 3.05
14	4.9 ± 0.2	3.2 ± 0.03
Selective GPBAR1 agonists		
15	3.7 ± 0.38	
17	2.54 ± 0.015	
20	6.8 ± 0.08	
21	5.9 ± 0.055	
24	0.91 ± 0.092	
25	7.6 ± 0.71	
27	1 ± 0.062	
29	4.9 ± 0.06	
30	1.98 ± 0.145	

^*^Data are mean ± SE of 3 experiments in duplicate.
