# Supplementary material for: Hyodeoxycholic acid derivatives as liver X receptor α and G-protein-coupled bile acid receptor agonists
Source: Sci Rep. 2017 Feb 24;7:43290. doi: 10.1038/srep43290 (PMC5324103; doi:10.1038/srep43290)

Supplementary information

Hyodeoxycholic acid derivatives as liver X receptor α and G-protein-coupled bile acid receptor agonists

Simona De Marino,[1] Adriana Carino,[2] Dario Masullo,[1] Claudia Finamore,[1] Silvia Marchianò,[2] Sabrina Cipriani,[2] Francesco Saverio Di Leva,[1] Bruno Catalanotti,[1] Ettore Novellino,[1] Vittorio Limongelli,[1,3] Stefano Fiorucci,[2,°] and Angela Zampella[1,°*]

[1]Department of Pharmacy, University of Naples "Federico II", Via D. Montesano, 49, I-80131 Naples, Italy
[2]Department of Surgery and Biomedical Sciences, Nuova Facoltà di Medicina, P.zza L. Severi 1-06132 Perugia, Italy
[3]UniversitàdellaSvizzeraItaliana (USI), Faculty of Informatics, Institute of Computational Science - Center for Computational Medicine in Cardiology, Via G. Buffi 13, CH-6900 Lugano, Switzerland.

Table of contents:

- Synthetic procedures S3
- Figure S1. FXR transactivation S15
- Figure S2. Binding pose of **14** S16
- Figure S3.The rmsd plot of the heavy atoms of **14** S17
- Figure S4. Binding pose of **14** in the LXRα LDB S18
- ^{1}H-NMR spectrum of compound **4** S19
- ^{1}H-NMR spectrum of compound **5** S20
- ^{1}H-NMR spectrum of compound **6** S21
- ^{1}H-NMR spectrum of compound **7** S22
- ^{1}H-NMR spectrum of compound **8** S23
- ^{1}H-NMR spectrum of compound **9** S24
- ^{1}H-NMR spectrum of compound **10** S25
- ^{1}H-NMR spectrum of compound **11** S26
- ^{1}H-NMR spectrum of compound **12** S27
- ^{1}H-NMR spectrum of compound **13** S28
- ^{1}H-NMR spectrum of compound **14** S29
- ^{1}H-NMR spectrum of compound **15** S30
- ^{1}H-NMR spectrum of compound **16** S31
- ^{1}H-NMR spectrum of compound **17** S32
- ^{1}H-NMR spectrum of compound **18** S33
- ^{1}H-NMR spectrum of compound **19** S34
- ^{1}H-NMR spectrum of compound **20** S35
- ^{1}H-NMR spectrum of compound **21** S36
- ^{1}H-NMR spectrum of compound **22** S37
- ^{1}H-NMR spectrum of compound **23** S38
- ^{1}H-NMR spectrum of compound **24** S39

- ^{1}H-NMR spectrum of compound **25** S40
- ^{1}H-NMR spectrum of compound **26** S41
- ^{1}H-NMR spectrum of compound **27** S42
- ^{1}H-NMR spectrum of compound **28** S43
- ^{1}H-NMR spectrum of compound **29** S44
- ^{1}H-NMR spectrum of compound **30** S45

Compound 34. To a solution of HDCA (6 g, 15.3 mmol) in 50 mL of dry methanol, *p*-toluenesulfonic acid (10 g, 58.1 mmol) was added at room temperature. After 1h, the mixture was quenched by addition of $NaHCO_3$ saturated solution until the neutrality and, after evaporation, the residue was extracted with EtOAc (3×100 mL). The combined extracts were washed with brine, dried on Na_2SO_4, and evaporated to give the methyl ester **31** in quantitative yield.

2,6-Lutidine (17.4 mL, 150 mmol) and *tert*-butyldimethylsilyltrifluoromethanesulfonate (10.3 mL, 45 mmol) were added at 0 °C to a solution of **31** (6.1 g, 15 mmol) in 50 mL of CH_2Cl_2. After 2 h stirring at 0°C, the reaction was quenched by addition of aqueous $NaHSO_4$ (1M, 100 mL). The layers were separated and the aqueous phase was extracted with CH_2Cl_2 (3×100 mL). The combined organic layers were washed with $NaHSO_4$, water, saturated aqueous $NaHCO_3$, brine and evaporated *in vacuo* to give protected methyl ester **32** in quantitative yield. To a solution of **32** (9.5 g, 15 mmol) in dry THF (50 mL) and dry methanol (1.82 mL, 45 mmol), $LiBH_4$ (22.5 mL, 2M in THF, 45 mmol) was added at 0 °C. After stirring for 2 h at 0 °C, the mixture was quenched by addition of NaOH 1M and then EtOAc. The organic phase was washed with water, dried (Na_2SO_4) and concentrated. Purification on silica gel (hexane/EtOAc9:1 and 0.5% TEA) gave alcohol **33** in 56% yield (5 g, 8.3 mmol). DMSO (6.6 mL, 92 mmol) was added dropwise for 15 min to a solution of oxalyl chloride (23 mL, 46mmol) in dry dichloromethane (50 mL) at -78 °C under argon atmosphere. After 30 min, a solution of alcohol **33**(4 g, 6.6 mmol) in dry CH_2Cl_2 was added via cannula and the mixture was stirred at -78 °C for 30 min. Et_3N (13.8 mL, 99 mmol) was added dropwise followed by NaCl saturated solution. The aqueous phase was extracted with Et_2O (3×100 mL). The combined organic phase was washed with water, dried (Na_2SO_4) and concentrated to give aldehyde **34** (4 g)as a colourless oil in quantitative yield.

Compound 4. To a solution of isopropyl triphenylphosphonium iodide (5.4 g, 12.5 mmol) in THF (2 mL), *n*-BuLi (5 mL, 12.5 mmol) was added dropwise at room temperature until the solution reached a red colour. After 30 min, a solution of aldehyde **34** (1.5 g, 2.5 mmol) in THF (5 mL) was added. After 1h, the mixture was quenched by addition of saturated aqueous $NaHCO_3$ (50 mL) and

extracted with EtOAc(3x50 mL). The organic phase was dried (Na_2SO_4) and concentrated. Purification on silica gel (hexane) gave **35** in 84% yield.

To a solution of **35** (1.3 g, 2.1 mmol) in MeOH, 1 mL of HCl 37% v/v was added. After 1h, silver carbonate was added, the reaction mixture was centrifuged, and the supernatant was concentrated *in vacuo*to give compound **4** as a colourless amorphous solid (850 mg, quantitative yield). An analytic sample was purified by HPLC on a Nucleodur 100-5 C18(5μm; 10 mm i.d. x 250 mm) with MeOH/H_2O (96:4) as eluent (flow rate 3 mL/min, t_R= 12 min). Selected ^1H NMR (400 MHz, $CDCl_3$): δ_H 5.10 (1H, t, *J* = 6.7 Hz, H-24), 4.08 (1H, dt, *J* = 11.9, 4.6 Hz, H-6β), 3.64 (1H, m, H-3β), 1.68 (3H, s, Me-26), 1.60 (3H, s, Me-27), 0.92 (3H, d, *J* = 7.0 Hz, Me-21), 0.91 (3H, s, Me-19), 0.65 (3H, s, Me-18); ^{13}C NMR (100 MHz, $CDCl_3$): δ_C 130.9, 125.3, 71.6, 68.2, 56.2 (x 2), 48.4, 42.9, 40.0, 39.9, 36.4, 36.2, 35.6, 35.3, 35.0, 34.9, 30.3, 29.3, 28.4, 25.7, 24.7, 24.3, 23.3, 20.9, 18.4, 17.7, 12.1. HR ESIMS *m/z* 403.3576 $[M+H]^+$, $C_{27}H_{46}O_2$ requires 403.3578.

Compound 5. A solution of **4** (350 mg, 0.87 mmol) in THF dry/MeOH dry (5 mL/5 mL, v/v) was hydrogenated in presence of $Pd(OH)_2$ 5% wt on activated carbon. After 12 h, the catalyst was filteredthrough Celite, and the recovered filtrate was concentrated under vacuum to give a colourless amorphous solid (350 mg, quantitative yield). An analytic sample was purified by HPLC on a Nucleodur 100-5 C18 (5μm; 10 mm i.d. x 250 mm) with MeOH/H_2O (95:5) as eluent (flow rate 3 mL/min, t_R= 8 min). Selected ^1H NMR (400 MHz, $CDCl_3$): δ_H 4.06 (1H, dt, *J* = 11.9, 4.6 Hz, H-6β), 3.62 (1H, m, H-3β), 0.91 (3H, s, Me-19), 0.90 (3H, d, *J* = 7.0 Hz, Me-21), 0.87 (3H, d, *J*= 6.6 Hz, Me-26), 0.86 (3H, d, *J*= 6.6 Hz, Me-27), 0.64 (3H, s, Me-18).^{13}C NMR (100 MHz, $CDCl_3$): δ_C 71.6, 68.2, 56.3, 56.2, 48.4, 42.9, 40.0, 39.9, 39.4, 36.4, 36.0, 35.9, 35.6, 35.5, 34.9, 30.4, 29.3, 28.4, 28.2, 22.7 (x 2), 24.4, 23.9, 23.3, 20.9, 18.8, 12.1.

HR ESIMS *m/z* 405.3733 $[M+H]^+$, $C_{27}H_{49}O_2$ requires 405.3735.

Compound 6.Compound **6** (141 mg, 0.34 mmol, 20%) was prepared from **34** (1 g, 1.65 mmol) by an analogous procedure to that detailed above for **4**.An analytic sample was purified by HPLC on a

Nucleodur 100-5 C18 (5μm; 4.6 mm i.d. x 250 mm) with $MeOH/H_2O$ (96:4) as eluent (flow rate 3 mL/min) gave compound **6** (t_R= 17.2 min).

Compound 7. Hydrogenation on **6** gave **7**.An analytic sample was purified by HPLC purificationon a Nucleodur 100-5 C18 (5μm; 4.6 mm i.d. x 250 mm) with $MeOH/H_2O$ (96:4) as eluent (flow rate 1 mL/min) gave compound **7** (t_R= 16 min).Selected ^1H NMR (400 MHz, CD_3OD): δ_H 4.01 (1H, dt, *J* = 11.9, 4.5 Hz, H-6β), 3.50 (1H, m, H-3β), 0.93 (3H, d, *J* = 6.6 Hz, Me-21), 0.92 (3H, s, Me-19), 0.87 (6H, d, *J* = 6.7 Hz, Me-27 and Me-28), 0.68 (3H, s, Me-18).^{13}C NMR (100 MHz, $CDCl_3$): δ_C71.6, 68.1, 56.2 (x 2), 48.4, 42.8, 39.9, 39.8, 39.1, 36.0, 35.9, 35.7, 35.5, 35.1, 34.8, 30.3, 29.1, 28.2, 28.0, 27.9, 26.3, 24.2, 23.7, 23.6, 23.5, 20.7, 18.6, 12.0.

HR ESIMS *m/z* 419.3889 $[M+H]^+$, $C_{28}H_{51}O_2$ requires 419.3892.

Compound 8.Compound **8**(370 mg, 0.99 mmol, 60%) was obtained from **34** (1 g, 1.65 mmol) by an analogous procedure to that detailed above for **4**. An analytic sample was purified by HPLC on a Nucleodur 100-5 C18 (5μm; 10 mm i.d. x 250 mm) with $MeOH/H_2O$ (96:4) as eluent (flow rate 3 mL/min)giving pure compound **8** (t_R= 5 min). Selected ^1H NMR (400 MHz, $CDCl_3$): δ_H 5.80 (1H, m, H-24), 4.99 (1H, d, *J* = 17.1 Hz, H-25), 4.91 (1H, d, *J* = 9.8 Hz, H-25), 4.05 (1H, dt, *J* = 12.0, 4.5 Hz, H-6β), 3.62 (1H, m, H-3β), 0.91 (3H, s, Me-19), 0.92 (3H, d, *J* = 7.0 Hz, Me-21), 0.64 (3H, s, Me-18).^{13}C NMR (100 MHz, $CDCl_3$): δ_C 139.6, 113.9, 71.6, 68.1, 56.2, 56.1, 48.4, 42.8, 39.9, 39.8, 36.0, 35.5, 35.3, 35.2, 35.1, 34.8, 30.5, 30.2, 29.2, 28.2, 24.2, 23.5, 20.7, 18.4, 12.0.

HR ESIMS *m/z* 375.3263 $[M+H]^+$, $C_{25}H_{43}O_2$ requires 375.3266.

Compound 9. Reduction of **8** (125 mg, 0.33 mmol) in the sameprocedure to that detailed above for **5**, gave 87 mg of compound **9** (70%). Purification by HPLC on a Nucleodur 100-5 C18 (5μm; 4.6 mm i.d. x 250 mm) with $MeOH/H_2O$ (9:1) as eluent (flow rate 1 mL/min), (t_R= 18.6 min) furnished pure compound **9**. Selected ^1H NMR (400 MHz, CD_3OD): δ_H3.99 (1H, dt, *J* = 12.2, 4.6 Hz, H-6β), 3.50 (1H, m, H-3β), 0.92 (3H, s, Me-19), 0.93 (3H, d, *J* = 6.8 Hz, Me-21), 0.90 (3H, t, *J* = 6.8 Hz, Me-25), 0.68 (3H, s, Me-18).^{13}C NMR (100 MHz, $CDCl_3$): δ_C 71.6, 68.1, 56.2, 56.1, 48.4, 42.8,

39.9, 39.8, 35.9, 35.7, 35.5, 35.2, 35.0, 34.8, 30.2, 29.2, 28.3, 28.2, 24.2, 23.5, 23.1, 20.7, 18.6, 14.2, 12.0.HR ESIMS *m/z* 377.3420 $[M+H]^+$, $C_{25}H_{45}O_2$ requires 377.3422.

Compounds 10 and **11.**Compound **36** (125 mg, 0.28 mmol, 67% over two steps), as a mixture of diasteroisomers at the side chain double bond, was prepared from **34** (250 mg, 0.41 mmol) by an analogous procedure to that detailed above for **4**. A small portion of the mixture was purified by HPLC on a Nucleodur 100-5 C18 (5μm; 10 mm i.d. x 250 mm) with MeOH/H_2O (96:4) as eluent (flow rate 3 mL/min) to give pure compounds **10** (t_R= 13.6 min) and **11** (t_R= 16.2 min).

Compound 10. Selected ^{1}H NMR (400 MHz, $CDCl_3$): δ_H 7.33-7.18(5H, Ph), 6.38 (1H, d, *J* = 15.7 Hz, H-25), 6.20 (1H, dt, *J* = 15.7, 6.8 Hz, H-24), 4.05 (1H, dt, *J* = 12.1, 4.5 Hz, H-6β), 3.62 (1H, m, H-3β), 2.28 (1H, m, H-23), 2.10 (1H, m, H-23), 0.96 (3H, d, *J* = 6.4 Hz, Me-21), 0.91 (3H, s, Me-19), 0.65 (3H, s, Me-18).^{13}C NMR (100 MHz, $CDCl_3$): δ_C138.0, 131.5, 129.3, 128.4, 126.8, 125.9, 71.6, 68.2, 56.2 (x 2), 48.4, 42.9, 40.0, 39.9, 36.4, 35.8, 35.6, 35.5, 35.2, 35.1, 30.4, 29.8, 29.3, 28.4, 24.4, 23.4, 20.9, 18.6, 12.1.HR ESIMS *m/z* 451.3576 $[M+H]^+$, $C_{31}H_{47}O_2$ requires 451.3580.

Compound 11. Selected ^{1}H NMR (400 MHz, $CDCl_3$): δ_H 7.33-7.21 (5H, Ph),6.39 (1H, d, *J* = 11.7 Hz, H-25), 5.64 (1H, dt, *J* = 11.7, 7.3 Hz, H-24), 4.05 (1H, dt, *J* = 12.0, 4.5 Hz, H-6β), 3.62 (1H, m, H-3β), 2.38 (1H, m, H-23), 2.22 (1H, m, H-23), 0.91(3H, s, Me-19), 0.89 (3H, d, *J* = 6.8 Hz, Me-21), 0.64 (3H, s, Me-18).^{13}C NMR (100 MHz, $CDCl_3$): δ_C 137.8, 133.6, 128.7, 128.6, 128.2, 126.4, 71.6, 68.2, 56.2 (x 2), 48.4, 42.9, 40.0, 39.9, 36.4, 36.2, 35.6, 35.5, 35.1 (x2), 30.4, 29.3, 28.4, 25.5, 24.4, 23.3, 20.9, 18.5, 12.1.

HR ESIMS *m/z* 451.3576 $[M+H]^+$, $C_{31}H_{47}O_2$ requires 451.3579.

Compound 12. Hydrogenation on **36** (40 mg, 0.09 mmol) gave compound **12** (quantitative yield). An analytic sample was purified by HPLC on a Nucleodur 100-5 C18 (5μm; 4.6 mm i.d. x 250 mm) with MeOH/H_2O (96:4) as eluent (flow rate 1 mL/min, t_R= 7.6 min). Selected ^{1}H NMR (400 MHz, $CDCl_3$): δ_H 7.27-7.16 (5H, Ph), 4.05 (1H, dt, *J* = 11.9, 4.5 Hz, H-6β), 3.62 (1H, m, H-3β), 2.60 (2H, m, H_2-25), 0.90 (3H, s, Me-19), 0.89 (3H, d, *J* = 6.8Hz, Me-21), 0.64 (3H, s, Me-18).^{13}C NMR (100

MHz, $CDCl_3$): δ_C 143.0, 128.4, 128.3, 125.6, 71.6, 68.2, 56.2 (x 2), 48.4, 42.9, 40.0, 39.9, 36.3, 36.1, 35.7, 35.6, 35.5, 35.2 (x 2), 32.0, 30.4, 29.3, 28.4, 26.0, 24.4, 23.4, 20.9, 18.6, 12.1.

HR ESIMS *m/z* 453.3733 $[M+H]^+$, $C_{31}H_{49}O_2$ requires 453.3735.

Compound 37.Compound **37 (**1.9 g, 4.8 mmol)was prepared as previously described.[34]

Compound 38. Aldehyde **38** (1 g, 1.7 mmol, 94%) was prepared from **37** (1.9 g, 4.8 mmol) by an analogous procedure to that detailed above for **34**.

Compound 13.Compound **13** (264 mg, 0.7 mmol, 80% over two steps) was preparedfrom **38** (500 mg, 0.85 mmol) by the same procedure described for **4**. An analytic sample was purified by HPLC on a Nucleodur 100-5 C18 (5μm; 10 mm i.d. x 250 mm) with $MeOH/H_2O$ (96:4) as eluent (flow rate 3mL/min, t_R= 16 min) giving pure compound **13**. Selected ^1H NMR (400 MHz, CD_3OD): δ_H 5.12 (1H, brt, J = 6.8 Hz, H-23), 4.01 (1H, dt, J = 11.9, 4.6 Hz, H-6β), 3.50 (1H, m, H-3β), 1.70 (3H, s, Me-26), 1.59 (3H, s, Me-25), 0.93 (3H, s, Me-19), 0.90 (3H, d, J = 6.7 Hz, Me-21), 0.69 (3H, s, Me-18).^{13}C NMR (100 MHz, CD_3OD): δ_C 132.8, 124.2, 72.4, 68.6, 57.6 (x 2), 49.9, 44.1, 41.3 (x 2), 38.2, 37.0, 36.8, 36.2, 35.6, 35.5, 31.1, 29.9, 29.5, 26.1, 25.5, 24.1, 21.9, 19.2, 18.0, 12.4.

HR ESIMS *m/z* 389.3420 $[M+H]^+$, $C_{26}H_{45}O_2$ requires 389.3424.

Compound 14. Hydrogenation on **13** (100 mg, 0.3 mmol), in the same procedure detailed for **5**, gave **14** (90 mg, 0.2 mmol, 90%). An analytic sample was purified by HPLC on a Nucleodur 100-5 C18 (5μm; 10 mm i.d. x 250 mm) with $MeOH/H_2O$ (96:4) as eluent (flow rate 3mL/min) giving pure compound **14** (t_R= 18.4 min). Selected ^1H NMR (400 MHz, CD_3OD): δ_H 4.01 (1H, dt, J = 12.1, 4.6 Hz, H-6β), 3.51 (1H, m, H-3β), 0.94 (3H, s, Me-19), 0.93(3H, d, J = 6.5 Hz, Me-21), 0.89 (3H, d, J = 6.0 Hz, Me-25), 0.87 (3H, d, J = 6.0 Hz, Me-26), 0.68 (3H, s, Me-18).^{13}C NMR was recorded on Varian Inova 100 MHz, using $CDCl_3$ as solvent: δ_C 71.6, 68.1, 56.2 (x 2), 48.4, 42.8, 39.9, 39.8, 35.9, 35.8, 35.5, 35.3, 35.1, 34.8, 33.5, 30.3, 29.2, 28.4, 28.2, 24.2, 23.5, 23.0, 22.4, 20.7, 18.7, 12.0.

HR ESIMS *m/z* 391.3576 $[M+H]^+$, $C_{26}H_{47}O_2$ requires 391.3579.

Compound 15. Wittig olefination on aldehyde **38** (500 mg, 0.85 mmol), as described for compound **4**, gave **15** (290 mg, 0.8 mmol, 95% over two steps). An analytic sample was purified by HPLC on a Nucleodur100-5 C18 (5μm; 10 mm i.d. x 250 mm) with MeOH/H_2O (96:4) as eluent (flow rate 3mL/min), giving pure compound **15** (t_R= 11.0 min).Selected ^{1}H NMR (400 MHz, $CDCl_3$): δ_H5.77 (1H, m, H-23), 5.01 (1H, ovl, H-24), 4.99 (1H, ovl, H-24), 4.06 (1H, dt, *J* = 12.1, 4.6 Hz, H-6β), 3.63(1H, m, H-3β), 0.92 (3H, d, *J* = 6.6 Hz, Me-21), 0.91 (3H, s, Me-19), 0.66 (3H, s, Me-18).^{13}C NMR (100 MHz, $CDCl_3$): δ_C 137.3, 115.7, 71.6, 68.1, 56.1, 55.8, 48.4, 42.8, 40.5, 39.9, 39.8, 35.9, 35.8, 35.5, 35.1, 34.8, 30.3, 29.2, 28.2, 24.2, 23.5, 20.7, 18.6, 12.0.

HR ESIMS *m/z* 361.3107 $[M+H]^+$, $C_{24}H_{41}O_2$ requires 361.3109.

Compound 16.Hydrogenation on **15** (100 mg,0.3mmol), in the same procedure detailed for **5**, gave **16** (88 mg, 0.2 mmol, 88%). Purification by HPLC on a Nucleodur100-5 C18 (5μm; 4.6 mm i.d. x 250 mm) with MeOH/H_2O (96:4) as eluent (flow rate 1 mL/min), gavepure compound **16** (t_R=8.0 min). Selected ^{1}H NMR (400 MHz, $CDCl_3$): δ_H 4.06 (1H, dt, *J* = 12.1, 4.6 Hz, H-6β), 3.62 (1H, m, H-3β), 0.91 (3H, s, Me-19), 0.89 (3H, d, *J* = 7.0 Hz, Me-21), 0.86 (3H, t, *J* = 7.6 Hz, Me-23), 0.64 (3H, s, Me-18).^{13}C NMR (100 MHz, $CDCl_3$): δ_C71.6, 68.2, 56.0, 55.9, 48.4, 42.7, 39.9, 39.8, 38.2,36.4, 35.5, 35.4, 35.1, 34.8, 30.2, 29.3,28.2, 24.3, 23.5, 20.8, 19.2, 18.6, 12.0, 14.5.

HR ESIMS *m/z* 363.3263 $[M+H]^+$, $C_{24}H_{43}O_2$ requires 363.3266.

Compound 39. To a solution of HDCA (2 g, 5.1 mmol) in dry pyridine (100 mL), an excess of acetic anhydride was added. Pyridine was concentrated under vacuum and the residue was poured into cold water (100 mL) and extracted with EtOAc (3×50 mL). The combined organic phases were dried (Na_2SO_4) and concentrated. Flash chromatography on silica gel using CH_2Cl_2/MeOH 98:2 as eluent gave **39** (2.35 g, quantitative yield).

Compound 40. Compound **39** (2.35 g, 4.9 mmol) was dissolved in toluene dry/pyridine dry (10 mL:100μL, 10:1 v/v) and $Cu(OAc)_2$ H_2O (2.9 g, 14.7 mmol) was added in dark. After 30 min,

$Pb(OAc)_4$ (11 g, 24.5 mmol) was added. After 3 h, the solution was heated to reflux for 1 h (no longer in the dark). The mixture was then cooled, and aqueous ethylene glycol was added. The resulting mixture was extracted with ether (3x50 mL) and the combined organic phases were washed with saturated solution of $NaHCO_3$, water and brine. After drying over Na_2SO_4, the residue was evaporatedunder vacuum to give **40**. Purification by flash chromatography on silica gel (Hexane/EtOAc 95:5 with 0.5% of TEA) gave pure **40** (360 mg, 0.83 mmol).

Compound 17. Compound **40** (360 mg, 0.83 mmol) was treated with CH_3ONa (224 mg, 4.15 mmol) in $CHCl_3$ dry/MeOH dry (10 mL:6mL, 5:3 v/v). After stirring for 12 h, water was added and methanol was evaporated. The residue was extracted with EtOAc (3x50mL). The combined organic layers were washed with brine, dried and evaporated to dryness to give 259 mg (90%) of **17**. An analytic sample was purified by HPLC on a Nucleodur 100-5 C18 (5μm; 4.6 mm i.d. x 250 mm) with $MeOH/H_2O$ (93:7) as eluent (flow rate 1 mL/min, t_R= 6 min). Selected ^1H NMR (400 MHz, $CDCl_3$): δ_H5.68 (1H, m, H-22), 4.89 (1H, d, J = 17.3 Hz, H-23), 4.81 (1H, d, J = 10.1 Hz, H-23), 4.06 (1H, dt, J = 11.8, 4.4 Hz, H-6β), 3.62 (1H, m, H-3β), 1.02 (3H, d, J = 6.5 Hz, Me-21), 0.91 (3H, s, Me-19), 0.66 (3H, s, Me-18).^{13}C NMR (100 MHz, $CDCl_3$): δ_C 145.3, 111.5, 71.6, 68.2, 56.3, 55.5, 48.5, 42.7, 41.2, 40.0, 39.8, 36.4, 35.6, 35.5, 34.9, 30.4, 29.3, 28.5, 24.2, 23.3, 20.9, 20.2, 12.1.

HR ESIMS *m/z* 347.2950 $[M+H]^+$, $C_{23}H_{39}O_2$ requires 347.2953.

Compound 18. Hydrogenation on **17** (100 mg, 0.29 mmol) furnished **18** (99 mg, 0.28 mmol, quantitative yield). An analytic sample was purified by HPLC on a Nucleodur 100-5 C18 (5μm; 4.6 mm i.d. x 250 mm) with $MeOH/H_2O$ (90:10) as eluent (flow rate 1 mL/min, t_R= 9.4 min). Selected ^1H NMR (400 MHz, $CDCl_3$): δ_H 4.07 (1H, dt, J = 12.1, 4.6 Hz, H-6β), 3.63 (1H, m, H-3β), 0.91 (3H, s, Me-19), 0.89 (3H, d, J = 7.0 Hz, Me-21), 0.82 (3H, t, J = 7.4 Hz, Me-23), 0.64 (3H, s, Me-18). ^{13}C NMR (100 MHz, $CDCl_3$): δ_C 71.6, 68.1, 56.1, 55.7, 48.4, 42.7, 39.9, 39.8, 37.2, 36.4, 35.5, 35.4, 35.1, 30.4, 29.3, 28.5, 28.4, 24.5, 23.5, 21.0, 18.0, 12.0, 10.4.

HR ESIMS *m/z* 349.3107 $[M+H]^+$, $C_{23}H_{41}O_2$ requires 349.3110.

Compound 41. Aldehyde **41** (1.8 g, 3.8 mmol, 72%) was preparedstarting from LCA (2 g, 5.3 mmol), as reported for aldehyde **34**.

Compound 19. Compound **19** (653 mg, 1.8 mmol, quantitative yield over two steps) was obtainedfrom **41** (900 mg, 1.9mmol) as described for **4**. An analytic sample was purified by HPLC on a Nucleodur 100-5 C18 (5μm; 4.6 mm i.d. x 250 mm) with MeOH/H_2O (99:1) as eluent (flow rate 1 mL/min, t_R= 10 min). Selected ^1H NMR (400 MHz, $CDCl_3$): δ_H5.80 (1H, m, H-24), 4.99 (1H, d, *J* = 17.1 Hz, H-25), 4.91 (1H, d, *J* = 9.8 Hz, H-25), 4.05 (1H, dt, *J* = 12.0, 4.5 Hz, H-6β), 3.62 (1H, m, H-3β), 0.91 (3H, s, Me-19), 0.92 (3H, d, *J* = 7.0 Hz, Me-21), 0.64 (3H, s, Me-18).^{13}C NMR (100 MHz, $CDCl_3$): δ_C 139.6, 113.9, 71.6, 56.5, 56.2, 42.7, 42.2, 40.5, 40.2, 36.5, 35.8, 35.3, 35.2, 34.8, 34.6, 30.5, 30.2, 28.3, 27.2, 26.4, 24.2, 23.5, 20.8, 18.4, 12.0.

HR ESIMS *m/z* 359.3314 $[M+H]^+$, $C_{25}H_{43}O$ requires 359.3317.

Compound 20. Hydrogenation on **19** (400 mg, 1.1 mmol) gave **20** (338 mg, 0.94 mmol, 86%). An analytic sample was purified byHPLC on a Nucleodur 100-5 C18 (5μm; 4.6 mm i.d. x 250 mm) with MeOH/H_2O (99:1) as eluent (flow rate 1 mL/min, t_R= 13.4 min). Selected ^1H NMR (400 MHz, $CDCl_3$): δ_H 3.63 (1H, m, H-3β), 0.93 (3H, s, Me-19), 0.92 (3H, ovl,Me-21), 0.89 (3H, t, *J*= 7.3 Hz, Me-25).^{13}C NMR (100 MHz, $CDCl_3$): δ_C 71.9, 56.5, 56.3, 42.7, 42.1, 40.5, 40.2, 36.5, 35.9, 35.7, 35.6, 35.3, 34.6, 30.6, 28.3 (x2), 27.2, 26.4, 24.2, 23.4, 23.1, 20.8, 18.6, 14.2, 12.0.

HR ESIMS *m/z* 361.3470 $[M+H]^+$, $C_{25}H_{45}O$ requires 361.3472.

Compound 21. Compound **21** (367 mg, 0.95 mmol, 40% over two steps) was prepared from **41** (900 mg, 1.9mmol) as detailed for **4**. An analytic sample was purified byHPLC on a Nucleodur 100-5 C18 (5μm; 4.6 mm i.d. x 250 mm) with MeOH/H_2O (99:1) as eluent (flow rate 1 mL/min, t_R= 12.4 min). Selected ^1H NMR (400 MHz, $CDCl_3$): δ_H5.08 (1H, t, *J* = 6.6 Hz, H-24), 3.62 (1H, m, H-3β), 1.68 (3H, s, Me-26), 1.60 (3H, s, Me-27), 0.92 (3H, ovl, Me-21), 0.92 (3H, s, Me-19), 0.64 (3H, s, Me-18).^{13}C NMR (100 MHz, $CDCl_3$): δ_C 130.9, 125.2, 71.9, 56.5, 56.2, 42.7, 42.1, 40.4,

40.2, 36.5, 36.1, 35.8, 35.6, 35.3, 34.6, 30.6, 28.3, 27.2, 26.4, 25.7, 24.7, 24.2, 23.4, 20.8, 18.6, 17.6, 12.0.

HR ESIMS *m/z* 387.3620 $[M+H]^+$, $C_{27}H_{47}O$ requires 387.3623.

Compound 22. Hydrogenation on **21** (100 mg, 0.26 mmol) gave **22** (98 mg, quantitative yield). An analytic sample was purified by HPLC on a Nucleodur 100-5 C18 (5μm; 4.6 mm i.d. x 250 mm) with MeOH/H_2O (99:1) as eluent (flow rate 1 mL/min, t_R=16.6 min). Selected ^1H NMR (400 MHz, $CDCl_3$): δ_H3.63 (1H, m, H-3β),0.93 (3H, s, Me-19), 0.91 (3H, d, *J*= 6.6 Hz, Me-21), 0.87 (6H, d, *J*= 6.7 Hz, Me-26 and Me-27), 0.65 (3H, s, Me-18).^{13}C NMR (100 MHz, $CDCl_3$): δ_C 71.9, 56.5, 56.3, 42.6, 42.0, 40.5, 40.2, 39.5, 36.5, 36.2, 35.9, 35.8, 35.3, 34.7, 30.6, 28.3 (x 2), 27.2, 26.4, 24.2, 23.8, 23.3, 22.5 (x 2), 20.8, 18.7, 12.1.

HR ESIMS *m/z* 389.3783 $[M+H]^+$, $C_{27}H_{49}O$ requires 389.3786.

Compound 42. Compound **42** (954 mg, 2.6 mmol, quantitative yield over two steps) was obtained from LCA (1 g, 2.7 mmol) as reported for compound **40**.

Compound 23. Compound **23** was obtained from **42** (954 mg, 2.6 mmol)as reported for **17**. Purification on silica gel (hexane/EtOAc97:3 and 0.5% TEA) gave pure **23** (228 mg, 0.70mmol,27% yield). Selected ^1H NMR (400 MHz, $CDCl_3$): δ_H5.68 (1H, m, H-22), 4.90 (1H, d, *J*= 17.2 Hz, H-23), 4.82 (1H, d, *J*= 10.0 Hz, H-23), 3.63 (1H, m, H-3β), 1.03 (3H, d, J= 6.2 Hz, Me-21), 0.93 (3H, s, Me-19), 0.68 (3H, s, Me-18).^{13}C NMR (100 MHz, $CDCl_3$): δ_C145.3, 111.5, 71.9, 56.4, 55.6, 42.7, 42.1, 41.2, 40.5, 40.1, 36.4, 35.9, 35.3, 34.6, 30.5, 28.5, 27.2, 26.4, 24.3, 23.4, 20.8, 20.2, 12.3.

HR ESIMS *m/z* 331.3001 $[M+H]^+$, $C_{23}H_{39}O$ requires 331.3004.

Compound 24. Hydrogenation on **23** (100 mg, 0.30mmol) gave **24** (22 mg, 0.07 mmol, 22%). HPLC purification on a Nucleodur 100-5 C18 (5μm; 4.6 mm i.d. x 250 mm) with MeOH/H_2O (92:8) as eluent (flow rate 1 mL/min,) gave pure compound **24** (t_R= 26 min). Selected ^1H NMR (400 MHz, $CDCl_3$): δ_H 3.62(1H, m, H-3β), 0.92(3H, s, Me-19), 0.89(3H, d, *J* = 6.6 Hz, Me-21),

0.83 (3H, t, J = 7.5 Hz, Me-23), 0.65 (3H, s, Me-18).^{13}C NMR (100 MHz, $CDCl_3$): δ_C71.8, 56.4, 56.2, 42.6, 42.0, 40.5, 40.2, 37.2, 36.5, 35.7, 35.3, 35.0, 30.6, 28.4, 28.3, 27.1, 26.4,24.2, 23.3, 20.8, 18.2, 12.1, 10.4.HR ESIMS *m/z* 333.3157 $[M+H]^+$, $C_{23}H_{41}O$ requires 333.3159.

Compound 43. Compound **43** was prepared as previously described.[35]

Compound 44. Compound **44** (820 mg, 1.7mmol, 34%) was prepared from **43** (2 g, 5.1 mmol) in the same operative conditions reported for aldehyde**34**.

Compound 25. Compound **25** (110 mg, 0.29 mmol, 34%) was prepared from **44** (400 mg, 0.84 mmol)in the same operative conditions reported for **4.** An analytic sample was purified by HPLC on a Nucleodur 100-5 C18 (5μm; 4.6 mm i.d. x 250 mm) with MeOH/H_2O (99:1) as eluent (flow rate 1 mL/min), giving pure compound **25** (t_R= 20.4 min). Selected ^{1}H NMR (400 MHz, $CDCl_3$): δ_H5.10 (1H, brt, *J* = 6.9 Hz, H-24), 3.63 (1H, m, H-3α), 1.69 (3H, s, Me-26), 1.61 (3H, s, Me-27), 0.94 (3H, d, *J* = 6.6 Hz, Me-21), 0.93 (3H, s, Me-19), 0.65 (3H, s, Me-18).^{13}C NMR (100 MHz, $CDCl_3$):δ_C 130.9, 125.2, 71.4, 56.5, 56.2, 54.4, 44.8, 42.6, 40.0, 38.2, 37.0, 36.1, 35.6, 35.5, 35.4, 32.0, 31.5, 28.7, 28.2, 25.7, 24.7, 24.2, 21.3, 18.6, 17.6, 12.3, 12.1.

HR ESIMS *m/z* 387.3627 $[M+H]^+$, $C_{27}H_{47}O$ requires 387.3630.

Compound 26. Hydrogenation on **25** (90 mg, 0.23 mmol) afforded **26** (86 mg, 0.22 mmol, quantitative yield). Purification by HPLC on a Nucleodur 100-5 C18 (5μm; 4.6 mm i.d. x 250 mm) with MeOH/H_2O (99:1) as eluent (flow rate 1 mL/min) gave pure compound **26** (t_R= 25.2 min). Selected ^{1}H NMR (400 MHz, $CDCl_3$): δ_H 3.61(1H, m, H-3α), 0.91 (3H, d, *J*= 6.5 Hz,Me-21), 0.87 (3H, d, *J*= 6.6 Hz, Me-26), 0.86 (3H, d, *J*= 6.6 Hz, Me-27), 0.81 (3H, s, Me-19), 0.66 (3H, s, Me-18).^{13}C NMR (100 MHz, $CDCl_3$): δ_C 71.4, 56.5, 56.3, 54.4, 44.9, 42.7, 40.0, 39.4, 38.2, 37.0, 36.4, 36.0, 35.7, 35.6, 32.1, 31.5, 28.7, 28.4, 28.2, 22.5 (x 2), 24.2, 23.9, 21.3, 18.6, 12.3, 12.1.

HR ESIMS *m/z* 389.3783 $[M+H]^+$, $C_{27}H_{49}O$ requires 389.3785.

Compound 27. Compound **27** (114 mg, 0.32 mmol, 38%) was obtained from **44** (400 mg, 0.84 mmol)in the same operative conditions reported for **4**. An analytic sample was purified by HPLC on

a Nucleodur 100-5 C18 (5μm; 4.6 mm i.d. x 250 mm) with MeOH/H_2O (99:1) as eluent (flow rate 1 mL/min), giving pure compound **27** (t_R= 15.4 min). Selected ^{1}H NMR (400 MHz, $CDCl_3$): δ_H 5.80 (1H, m, H-24), 4.98 (1H, d, *J* = 17.0Hz, H-25), 4.90 (1H, d, *J* = 9.8 Hz, H-25), 3.60(1H, m, H-3α), 0.92 (3H, d, *J* = 7.0 Hz, Me-21), 0.80 (3H, s, Me-19), 0.65 (3H, s, Me-18).^{13}C NMR (100 MHz, $CDCl_3$):δ_C 139.6, 113.9, 71.5, 56.5, 56.3, 54.3, 44.8, 42.8, 39.9, 38.2, 36.9, 35.7, 35.6, 35.2, 34.8, 32.1, 31.5, 30.2, 28.7, 28.2, 24.1, 21.3, 18.6, 12.3, 12.1.

HR ESIMS *m/z* 359.3314 $[M+H]^+$, $C_{25}H_{43}O$ requires 359.3317.

Compound 28. Hydrogenation on **27** (100 mg, 0.28 mmol) gave **28** (98 mg, 0.27 mmol, quantitative yield). Purification by HPLC on a Nucleodur 100-5 C18 (5μm; 4.6 mm i.d. x 250 mm) with MeOH/H_2O (99:1) as eluent (flow rate 1 mL/min), gave pure compound **28** (t_R= 19.4 min). Selected ^{1}H NMR (400 MHz, $CDCl_3$): δ_H 3.63 (1H, m, H-3α), 0.88 (3H, ovl, Me-21), 0.88 (3H, ovl, Me-25),0.80 (3H, s, Me-19), 0.65 (3H, s, Me-18).^{13}C NMR (100 MHz, $CDCl_3$):δ_C 71.6, 56.4, 56.2, 54.3, 45.0, 42.7, 39.9, 38.3, 36.9, 35.9, 35.7, 35.6, 35.3, 32.1, 31.5, 28.6, 28.4, 28.2, 24.2, 23.1, 21.2, 18.6, 14.2, 12.3, 12.0.

HR ESIMS *m/z* 361.3470 $[M+H]^+$, $C_{25}H_{45}O$ requires 361.3473.

Compound 45. Compound **43** (1 g, 2.6 mmol) was hydrolyzed with NaOH (96 mg, 2.4 mmol) in a solution of MeOH:H_2O 1:1 v/v (10 mL). The mixture was stirred for 4 h at reflux. The resulting solution was then acidified with HCl 6N and extracted with ethyl acetate (3x50 mL). The collected organic phases were washed with brine, dried over Na_2SO_4 anhydrous and evaporated under reduced pressure to give the carboxylic acid. The intermediate was treatedin the same operative conditions reported for **40** furnishing compound **45** (755 mg, 2.02 mmol, 78% over three steps).

Compound 29. Compound **29** (413 mg, 1.25 mmol, 62%) was obtained from**45** (755 mg, 2.02 mmol) in the same operative conditions reported for **17**. An analytic sample was purified by HPLC on a Nucleodur 100-5 C18 (5μm; 4.6 mm i.d. x 250 mm) with MeOH/H_2O (96:4) as eluent (flow rate 1 mL/min), giving pure compound **29** (t_R= 17.4 min). Selected ^{1}H NMR (400 MHz, CD_3OD):

δ_H5.66 (1H, m, H-22), 4.89 (1H, d, J = 17.4 Hz, H-23), 4.81 (1H, ovl with solvent signal, H-23), 3.50 (1H, m, H-3α), 1.02 (3H, d, J = 6.5 Hz, Me-21), 0.83 (3H, s, Me-19), 0.71 (3H, s, Me-18).^{13}C NMR (100 MHz, $CDCl_3$): δ_C 145.3, 111.6, 71.4, 56.4, 55.5, 54.4, 45.0, 42.7, 41.2, 39.9, 38.2, 36.9, 35.6, 35.5, 32.1, 31.5, 28.7, 28.3, 24.2, 21.3, 20.2, 12.3, 12.1.

HR ESIMS *m/z* 331.3001 $[M+H]^+$, $C_{23}H_{39}O$ requires 331.3003.

Compound 30. Hydrogenation on **29** (300 mg, 0.9 mmol) gave **30** (287 mg, 0.87 mmol, quantitative yield). Purification by HPLC on a Nucleodur 100-5 C18 (5μm; 4.6 mm i.d. x 250 mm) with MeOH/H_2O (99:1) as eluent (flow rate 1 mL/min),gave pure compound **30** (t_R= 14.8 min). Selected ^{1}H NMR (400 MHz, $CDCl_3$): δ_H 3.63 (1H, m, H-3α), 0.93 (3H, s, Me-19), 0.90 (3H, d, J = 6.7 Hz, Me-21), 0.83 (3H, t, J = 7.5 Hz, Me-23), 0.65 (3H, s, Me-18).^{13}C NMR (100 MHz, $CDCl_3$): δ_C71.4, 56.5, 56.0, 54.4, 44.9, 42.6, 40.2, 38.2, 37.2, 36.9, 35.8, 35.5, 32.0, 31.5, 28.7, 28.5, 28.3, 24.1, 21.3, 18.2, 12.2, 12.0, 10.4.

HR ESIMS *m/z* 333.3157 $[M+H]^+$, $C_{23}H_{41}O$ requires 333.3159.

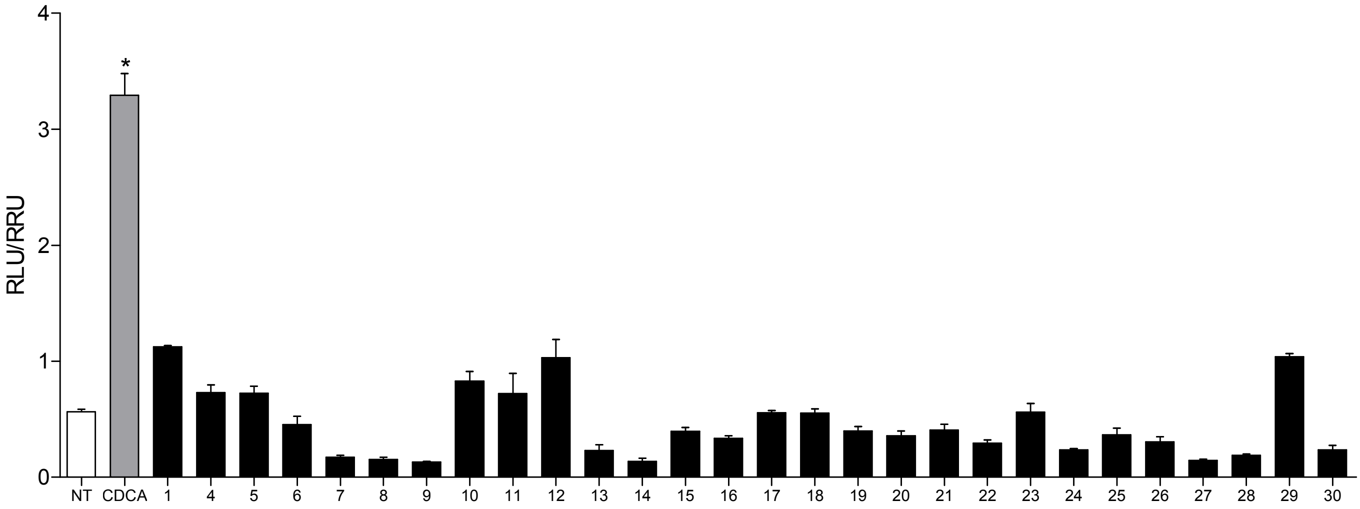

Figure S1.**Agonism on FXR by transactivation assay.** HepG2 cells were transfected with pSG5-FXR, pSG5-RXR, Pgl4.70-Renilla, and p(hsp27)TKLUC vectors. Cells were stimulated with HDCA (**1**) and compounds **4-30** (10 μM). CDCA (10 μM) was used as a positive control. Results are expressed as mean ± SE; *p<0.05 *versus* not treated cells (NT).

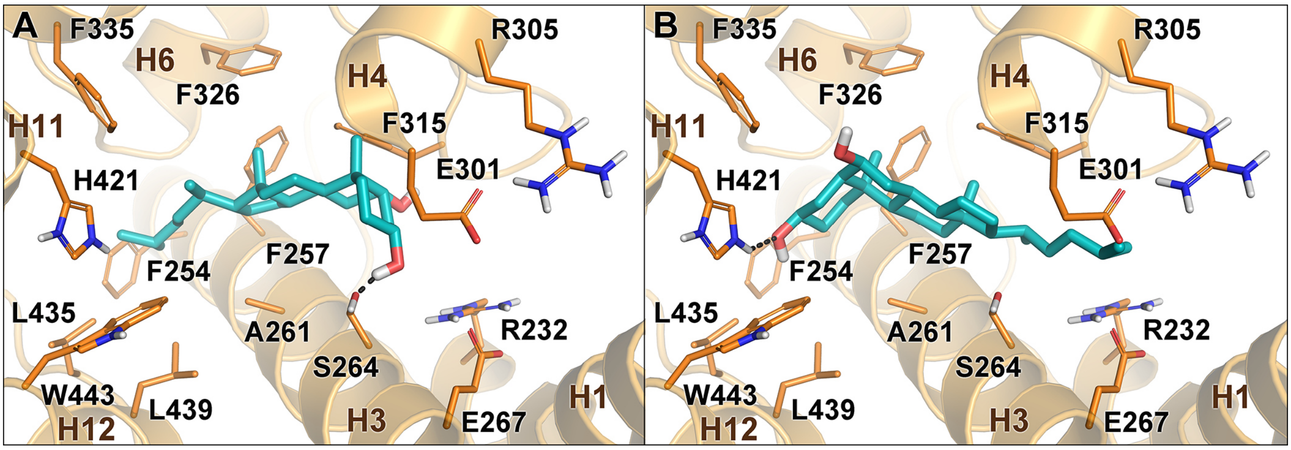

Figure S2. Binding poses A (A) and B (B) of **14**(cyan sticks) in the LXRαLDB (PDB code: 3IPU)as predicted by docking calculations. LXRαis shown as orange cartoons. Amino acids important for ligand binding are highlighted as sticks. Non-polar hydrogens are omitted for clarity.

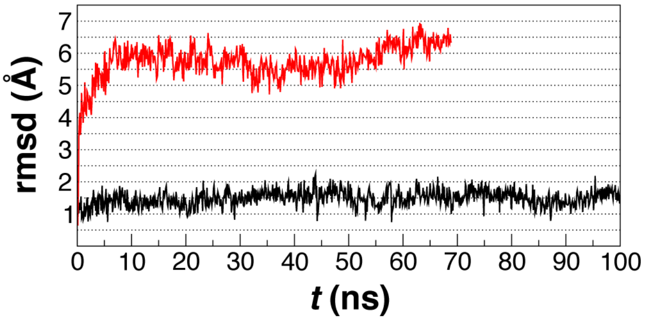

Figure S3. The rmsd plot of the heavy atoms of **14** during the MD simulations ofbinding modes A (black) and B (red) to LXRα. Prior to the rmsd calculations, trajectory frames were aligned using the coordinates of the Cα carbons of the receptor helices.Since binding mode B showed early to be unstable, its simulation was stopped at 70 ns.

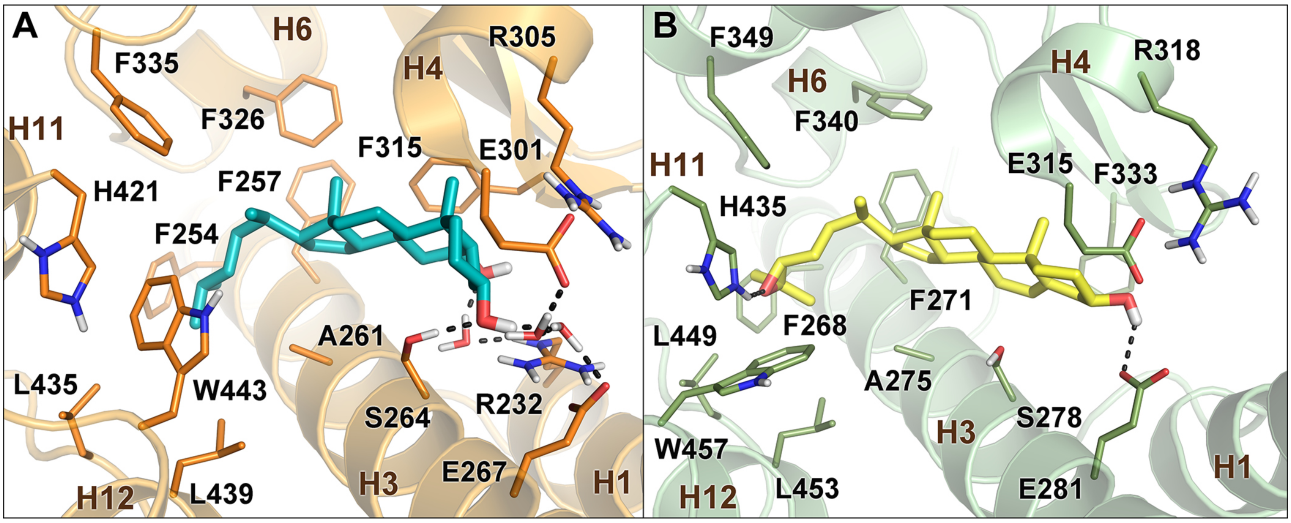

Figure S4. (A) Binding pose of **14**(cyan sticks) in the LXRα LDB (PDB code: 3IPU) as predicted by MD simulations. (B) Crystallographic pose of 24(*S*),25-epoxycholesterol (yellow sticks) in the LXRβ LDB (PDB code: 1P8D).LXRαand LXRβare shown as orange and greencartoons, respectively. Amino acids important for ligand binding are highlighted as sticks. Non-polar hydrogens are omitted for clarity

.

^{1}H NMR (400 MHz, $CDCl_3$) of compound **4**

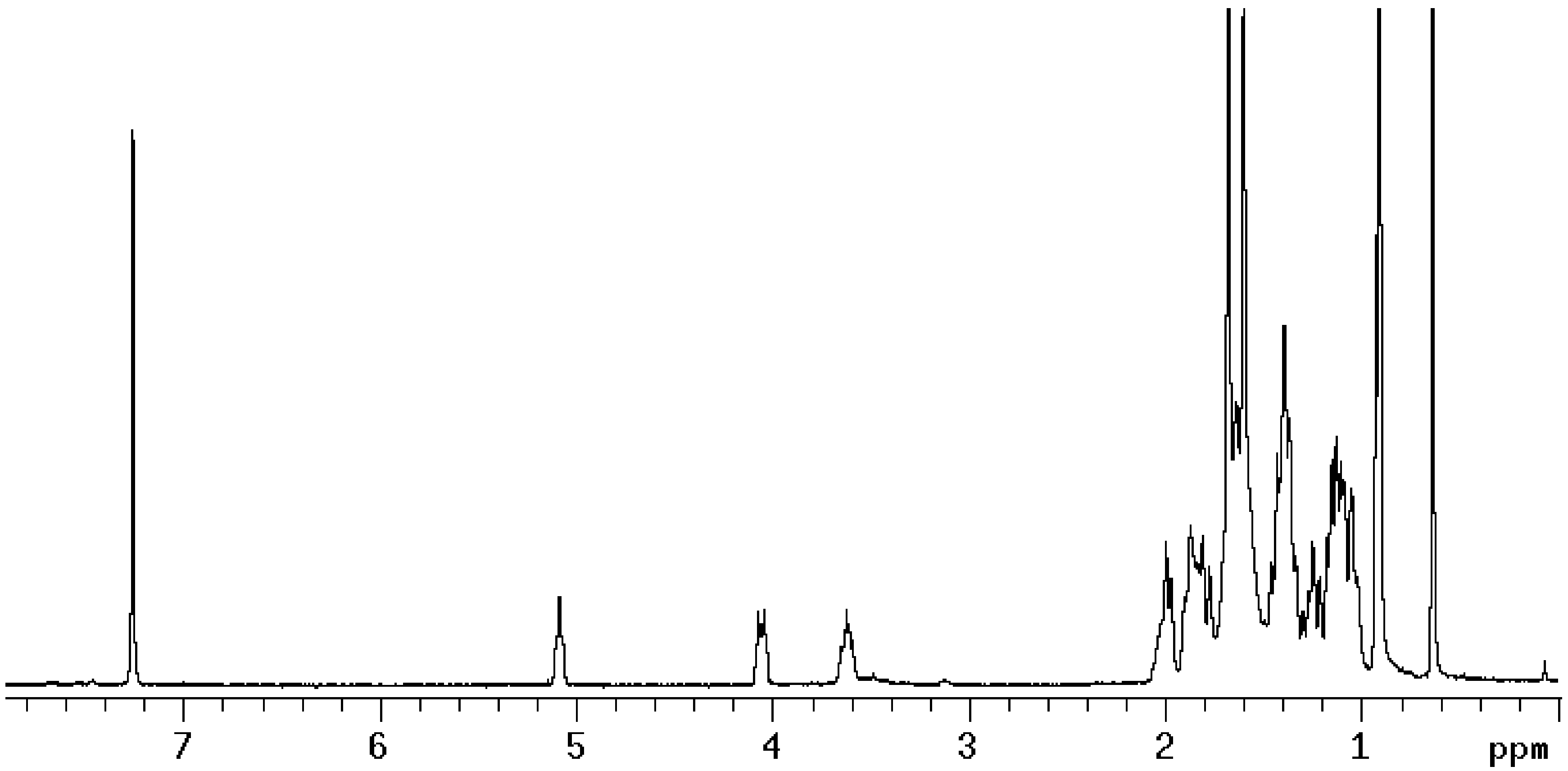

^{1}H NMR (400 MHz, $CDCl_3$) of compound **5**

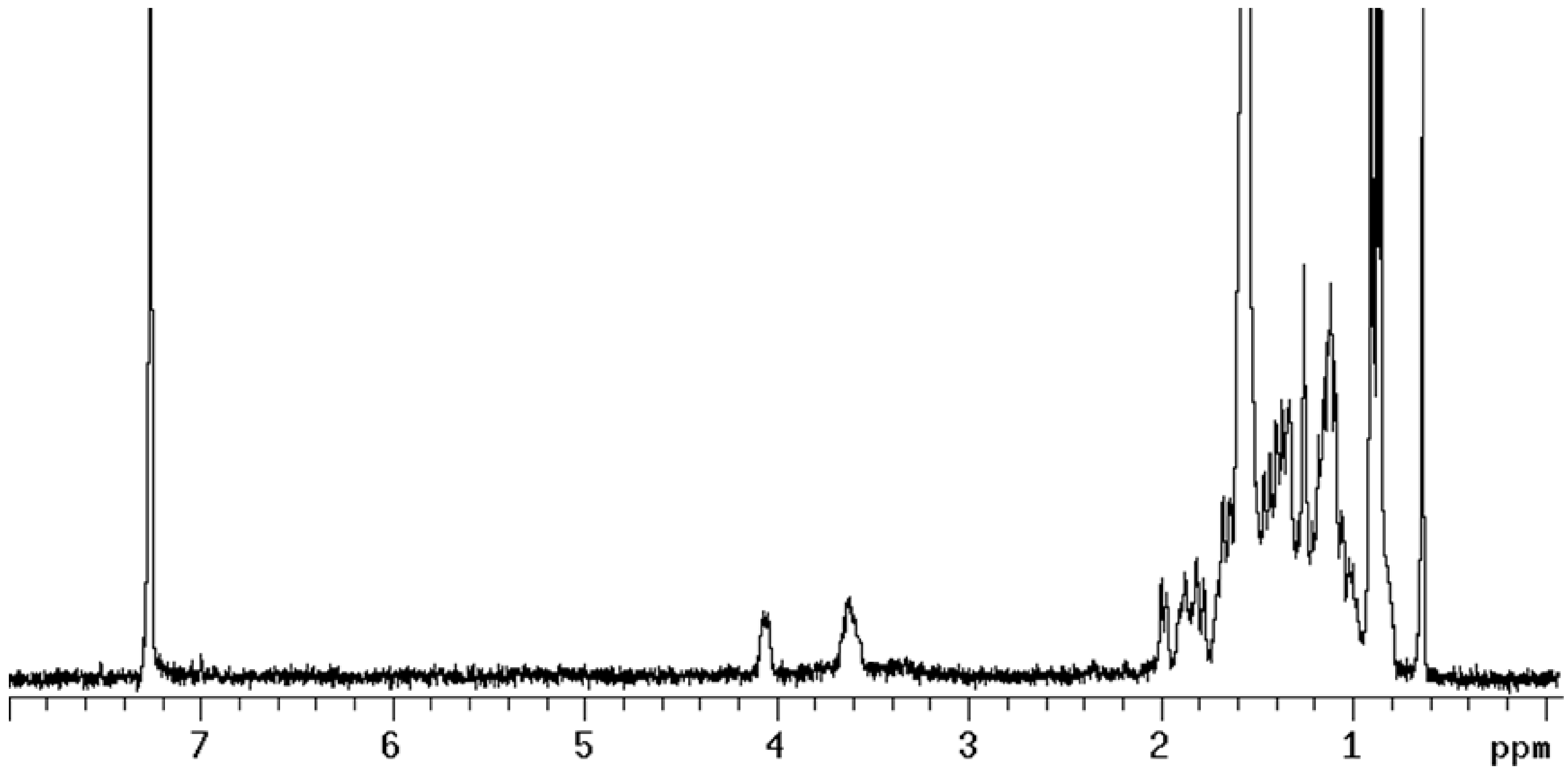

^{1}H NMR (400 MHz, $CDCl_3$) of compound **6**

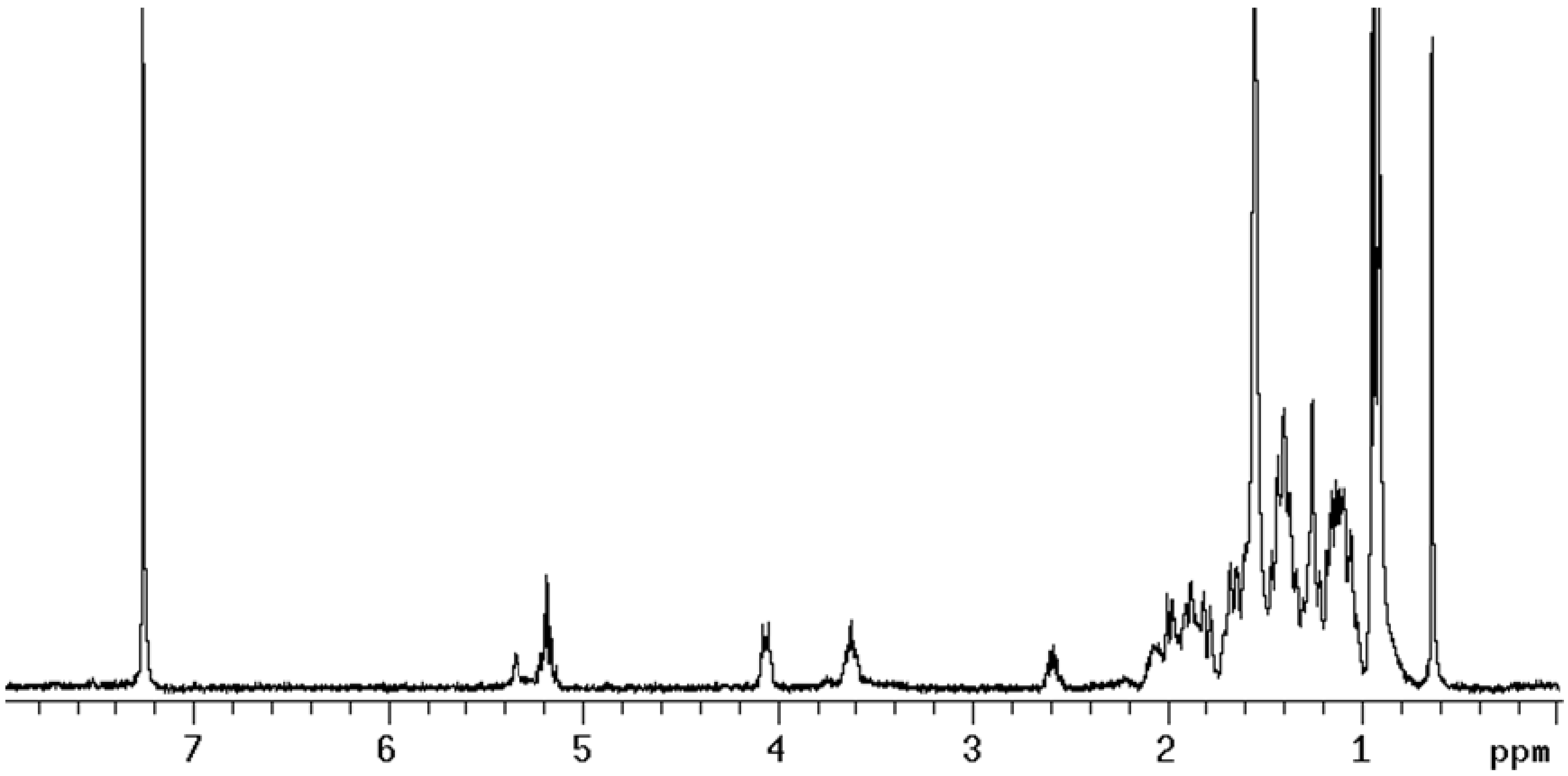

^{1}H NMR (400 MHz, CD_3OD) of compound **7**

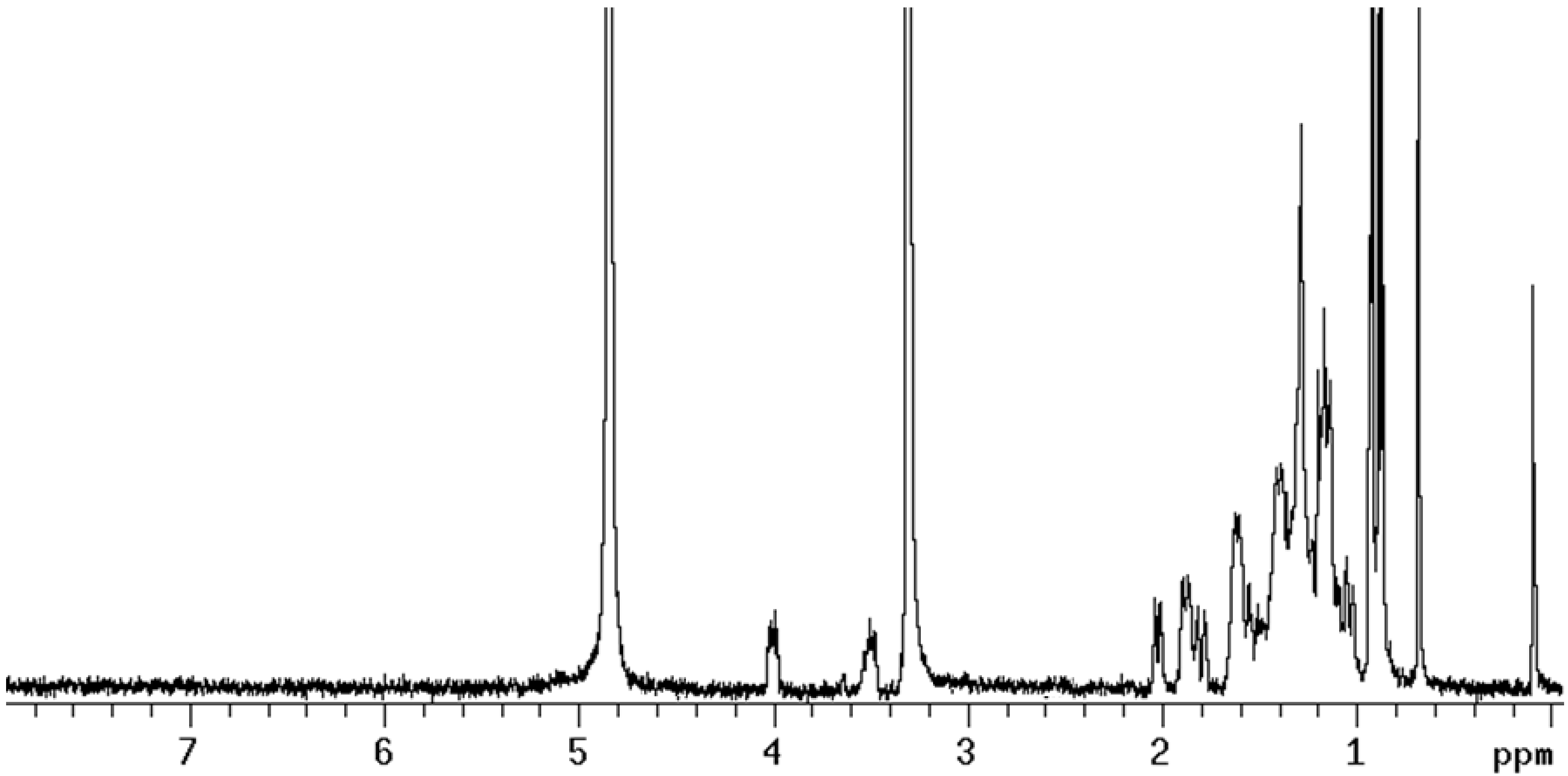

^{1}H NMR (400 MHz, $CDCl_3$) of compound **8**

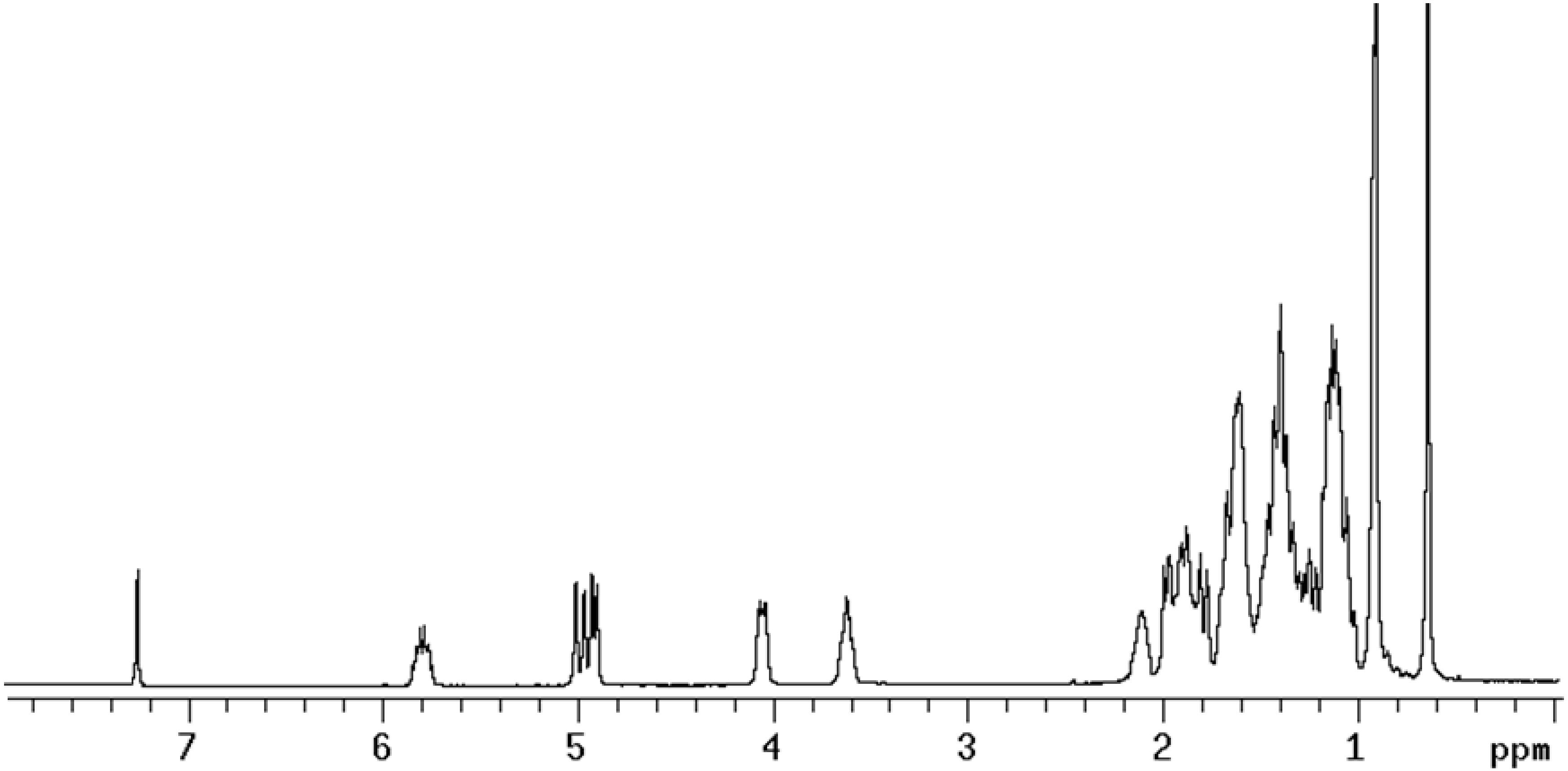

^{1}H NMR (400 MHz, CD_3OD) of compound **9**

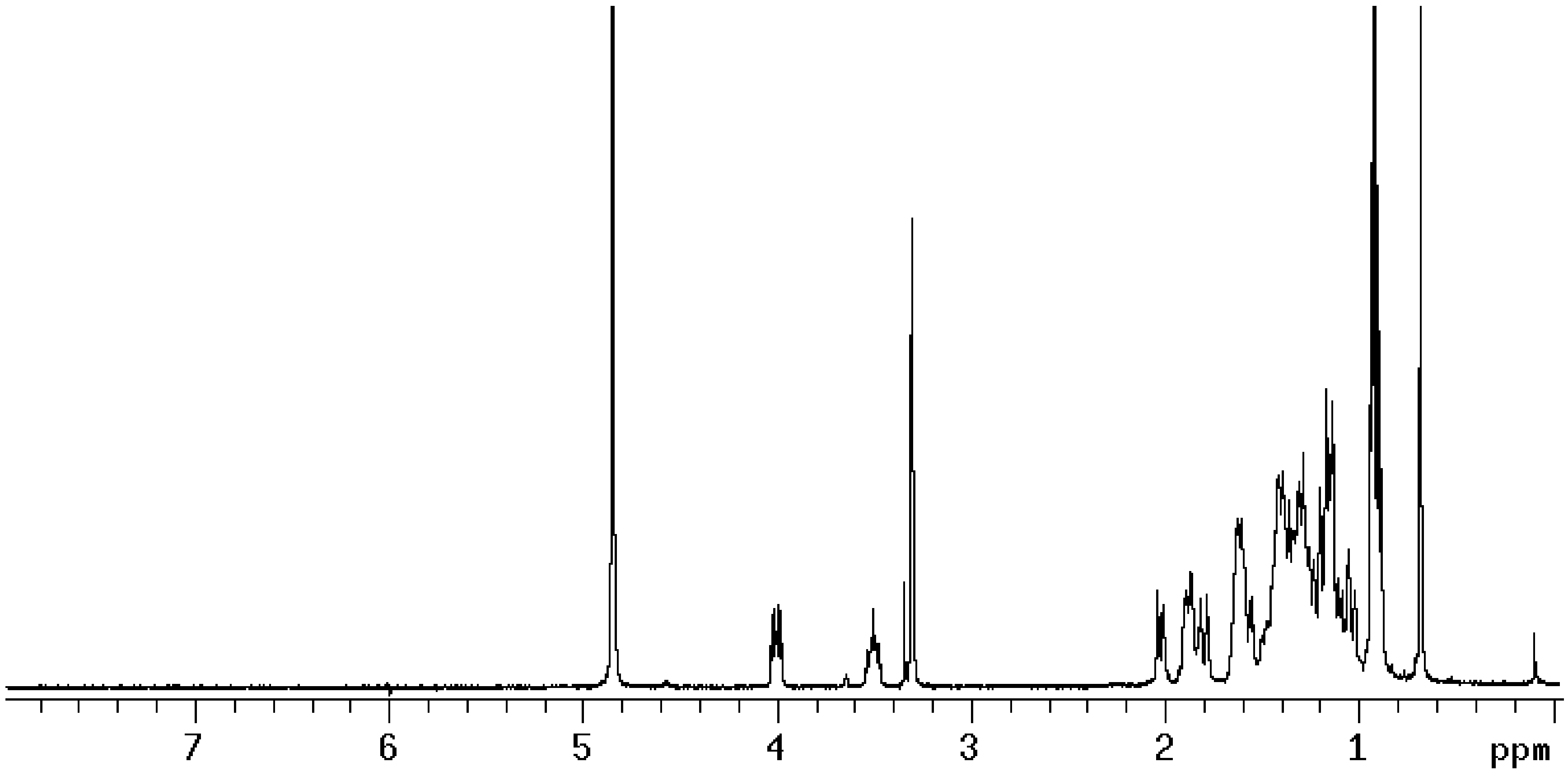

^1H NMR (400 MHz, $CDCl_3$) of compound **10**

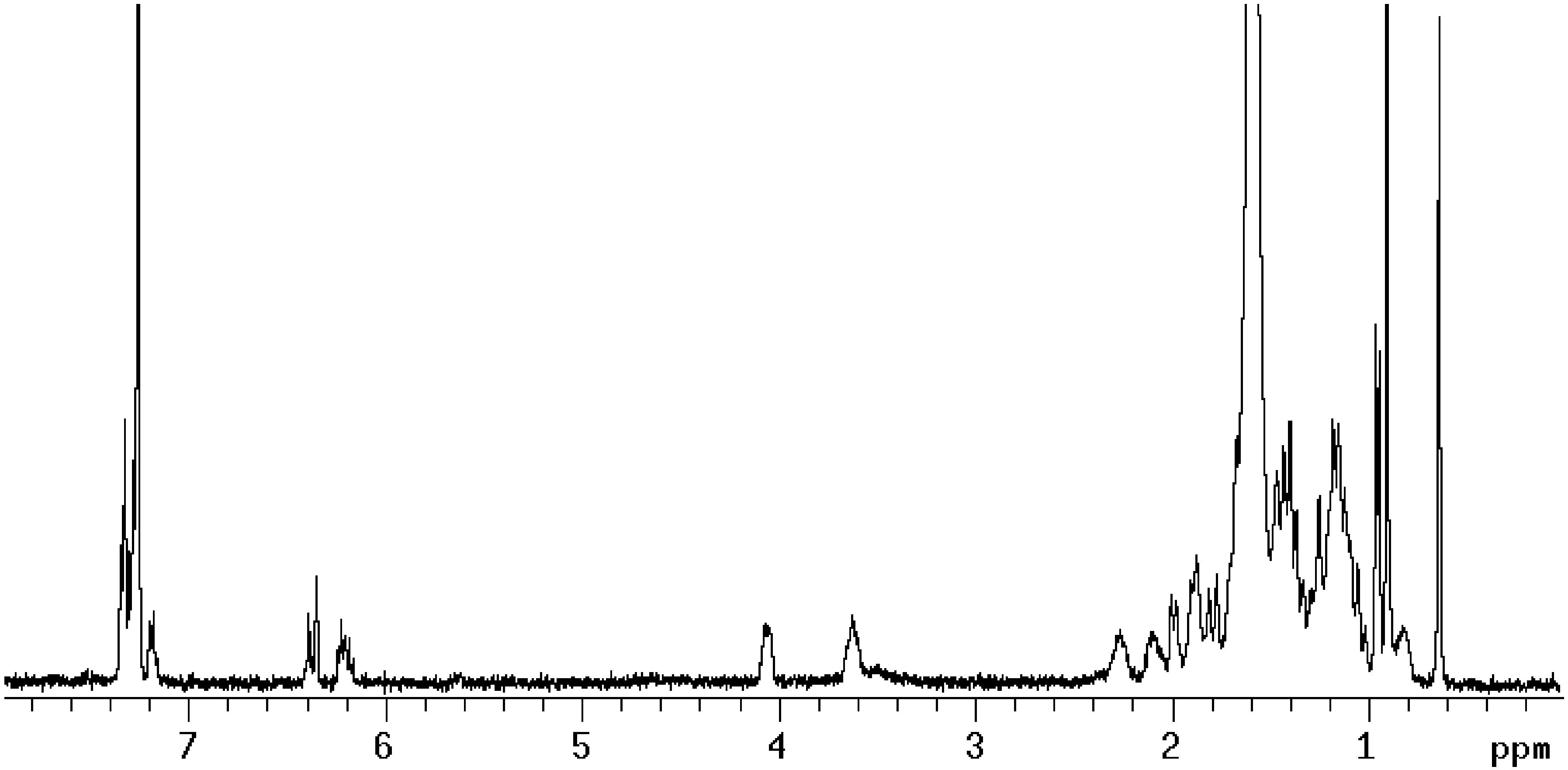

^{1}H NMR (400 MHz, $CDCl_3$) of compound **11**

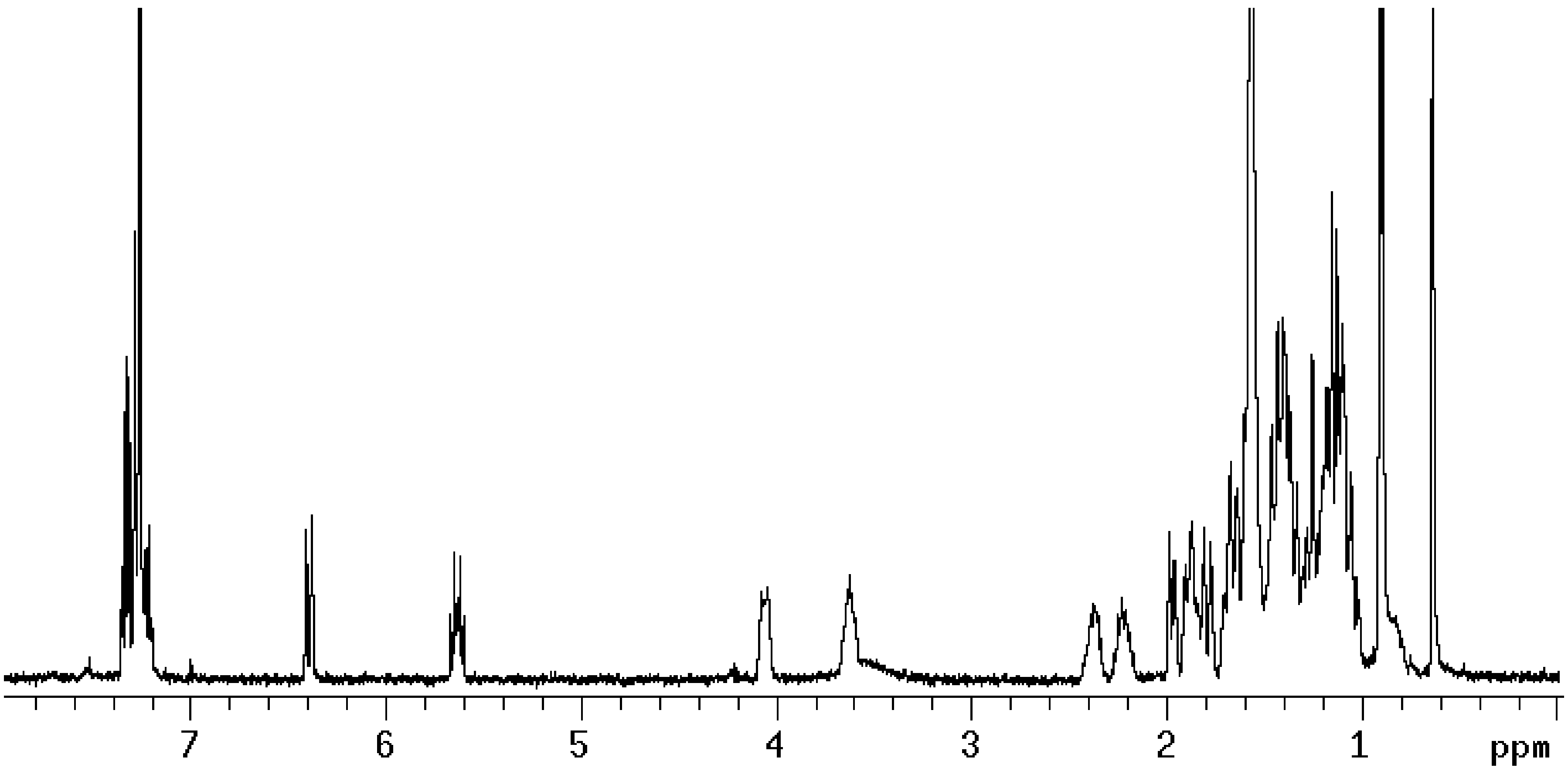

^{1}H NMR (400 MHz, $CDCl_3$) of compound **12**

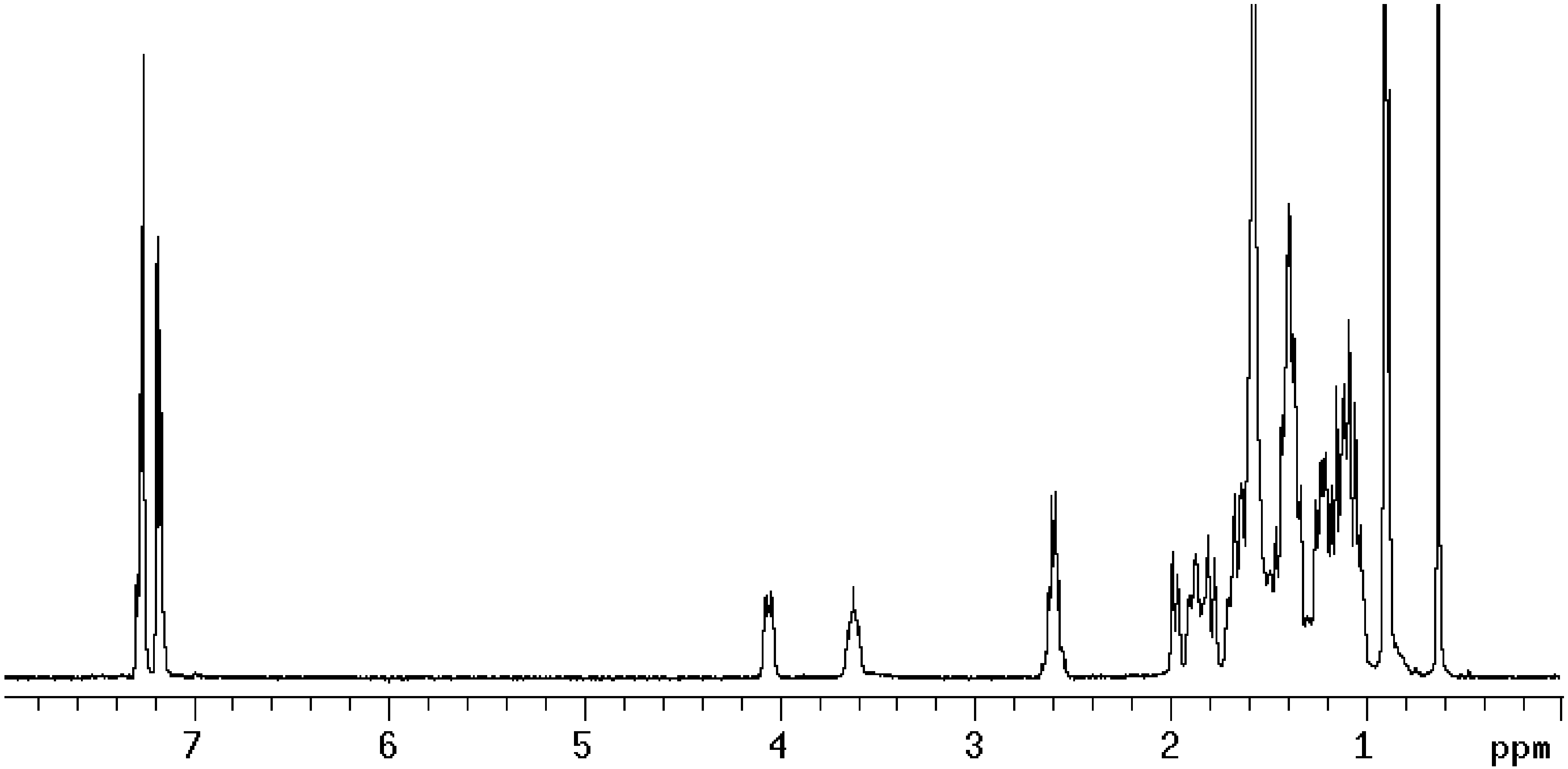

^{1}H NMR (400 MHz, CD_3OD) of compound **13**

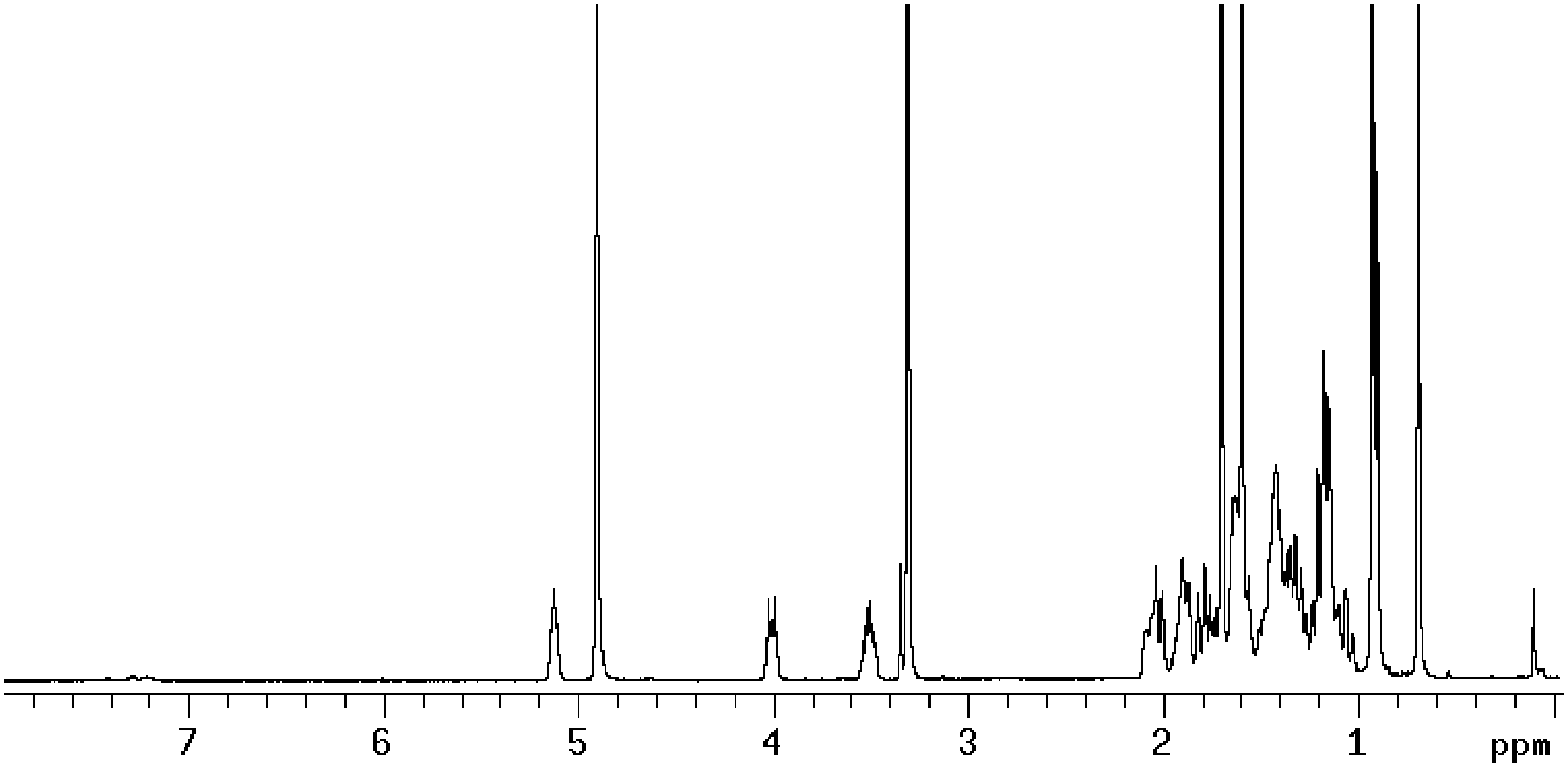

^{1}H NMR (400 MHz, CD_3OD) of compound **14**

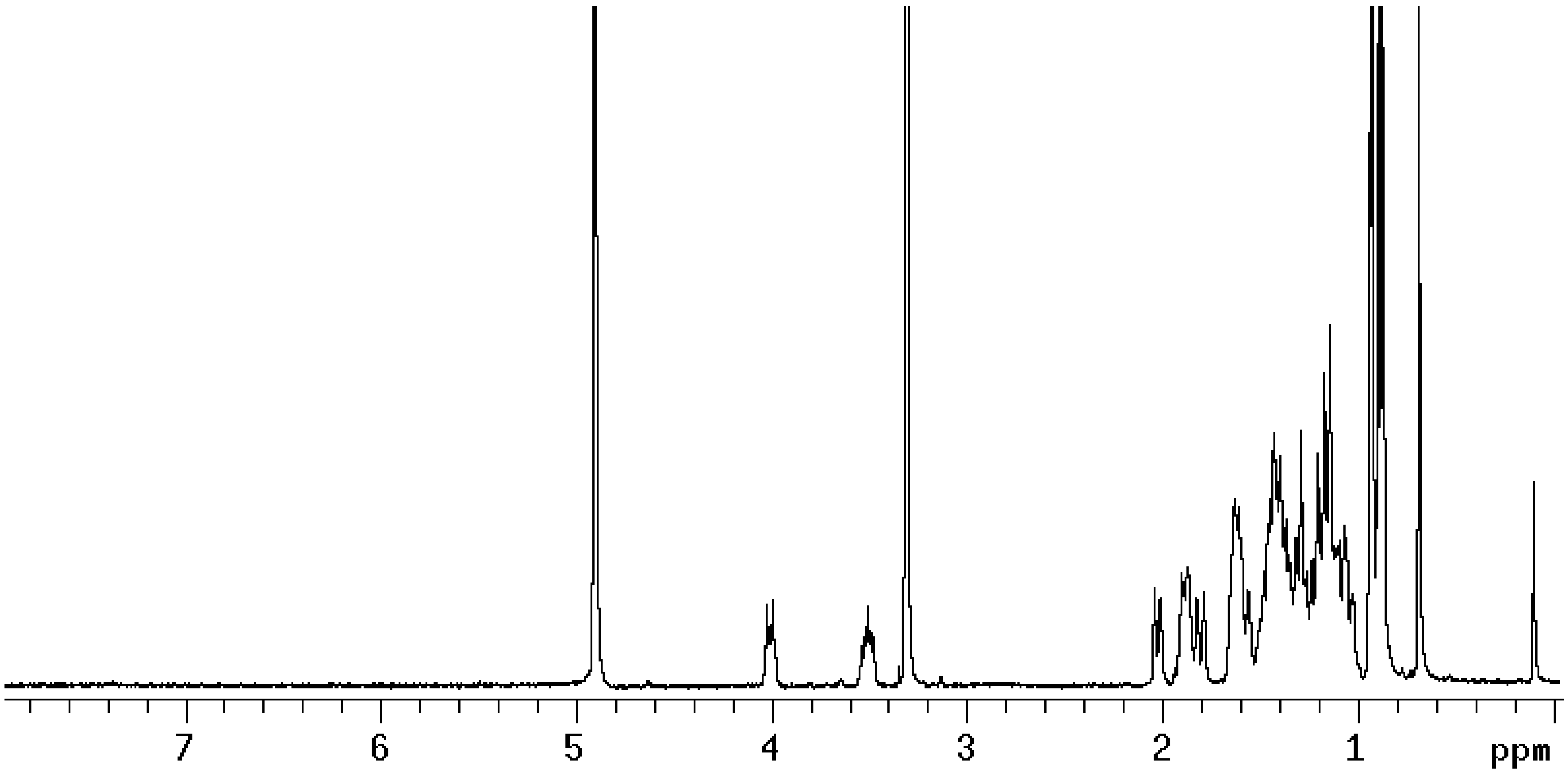

^1H NMR (400 MHz, $CDCl_3$) of compound **15**

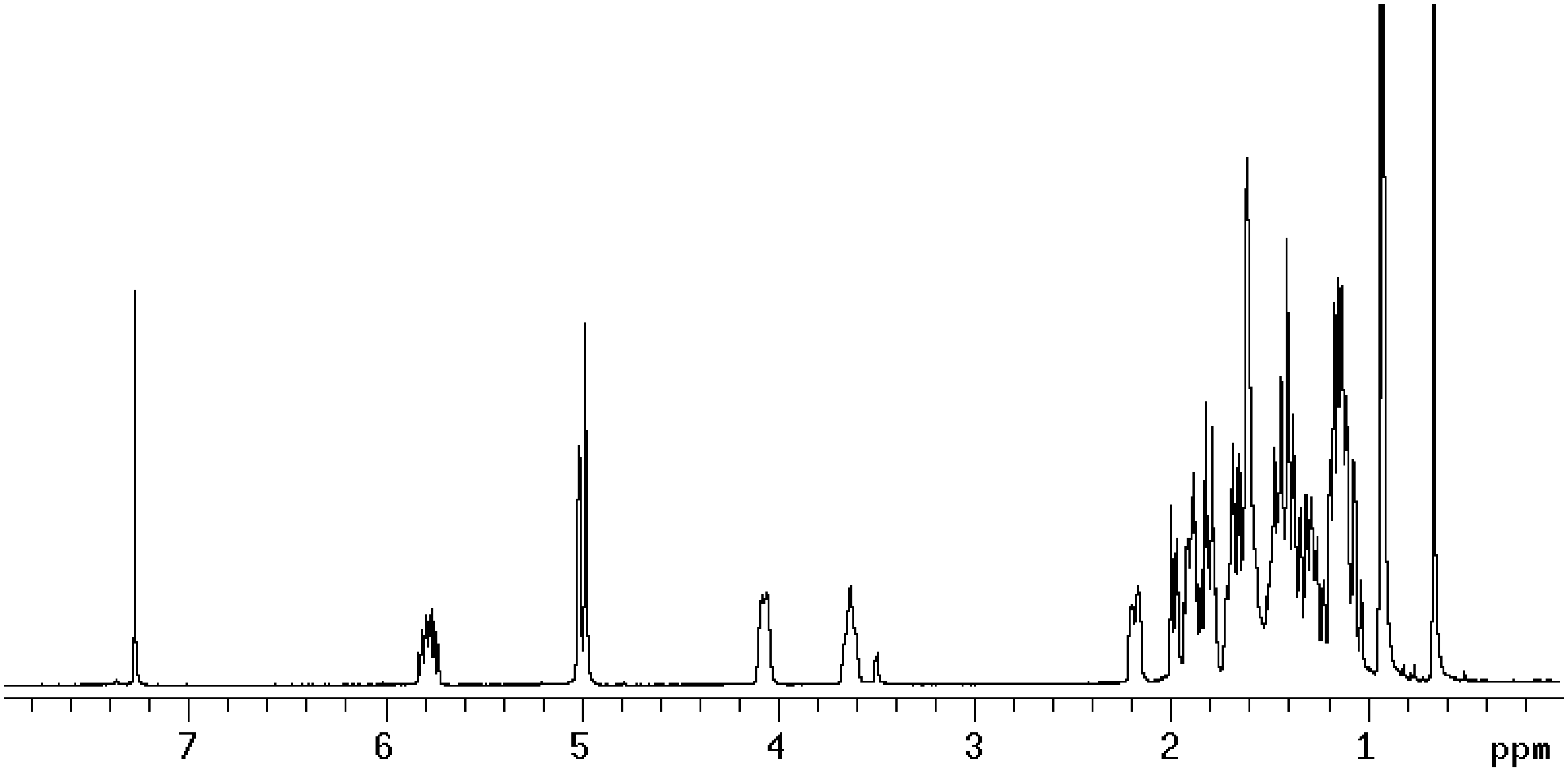

^{1}H NMR (400 MHz, $CDCl_3$) of compound **16**

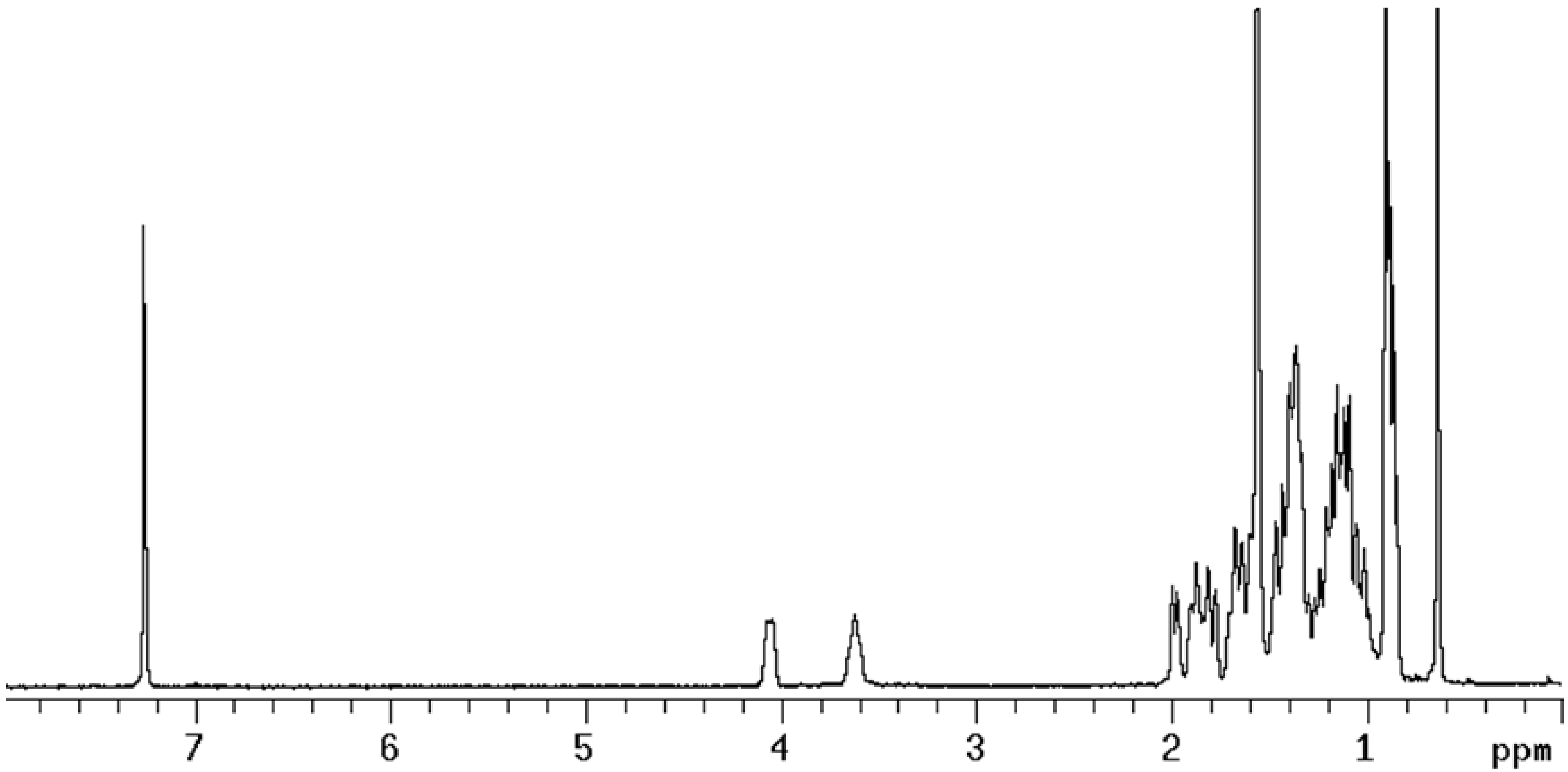

^{1}H NMR (400 MHz, $CDCl_3$) of compound **17**

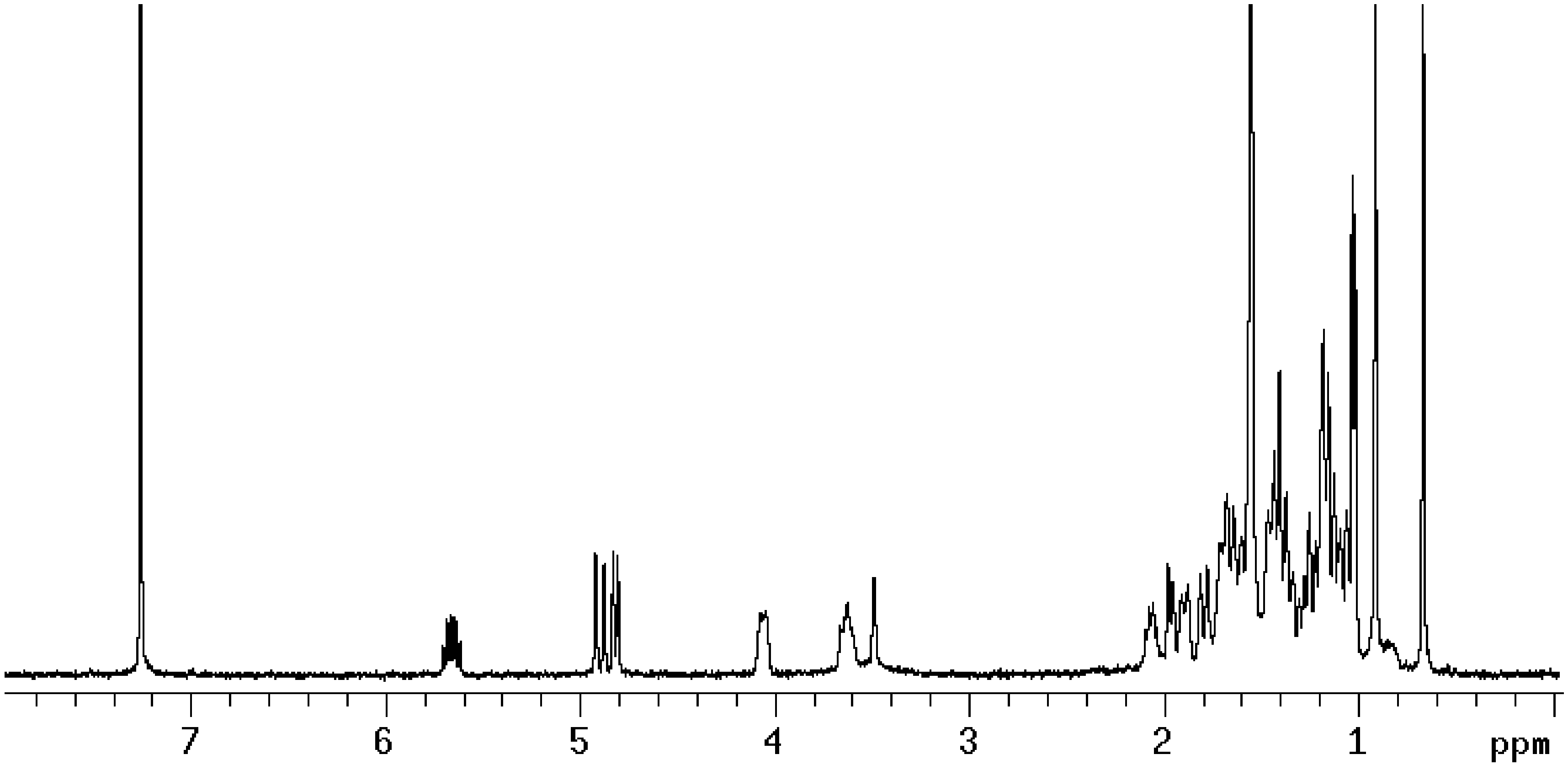

^{1}H NMR (400 MHz, $CDCl_3$) of compound **18**

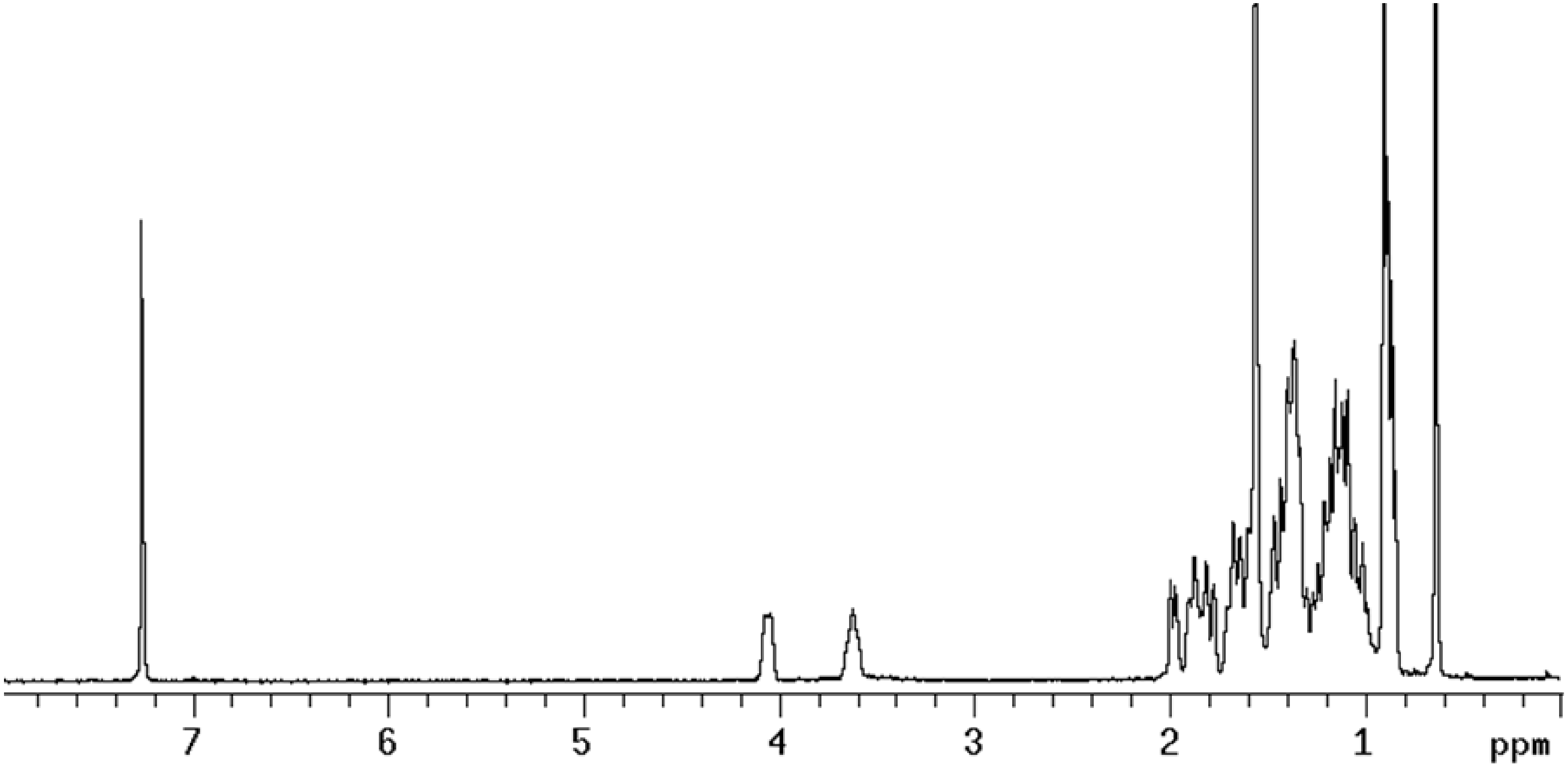

^{1}H NMR (400 MHz, $CDCl_3$) of compound **19**

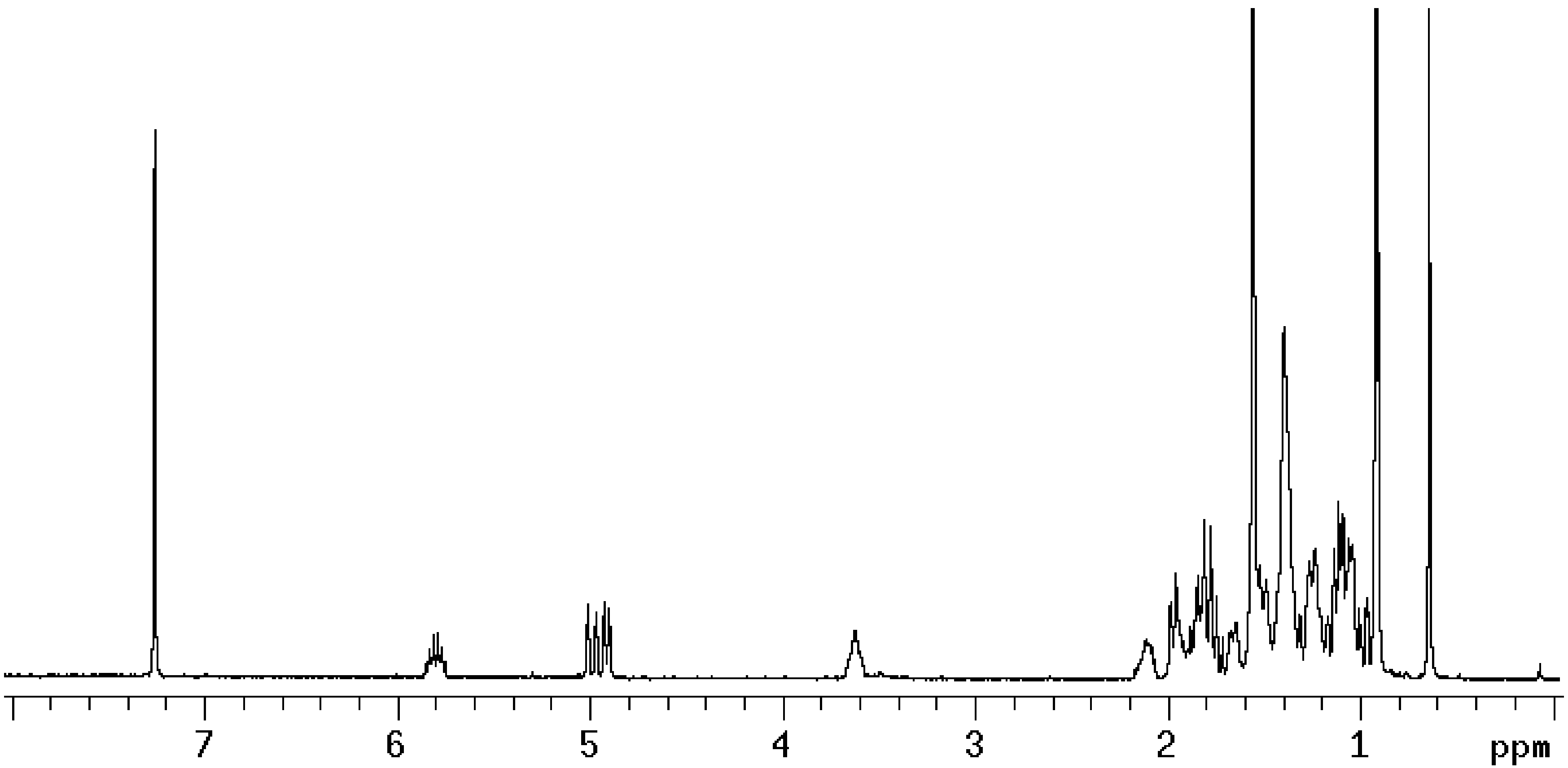

^1H NMR (400 MHz, $CDCl_3$) of compound **20**

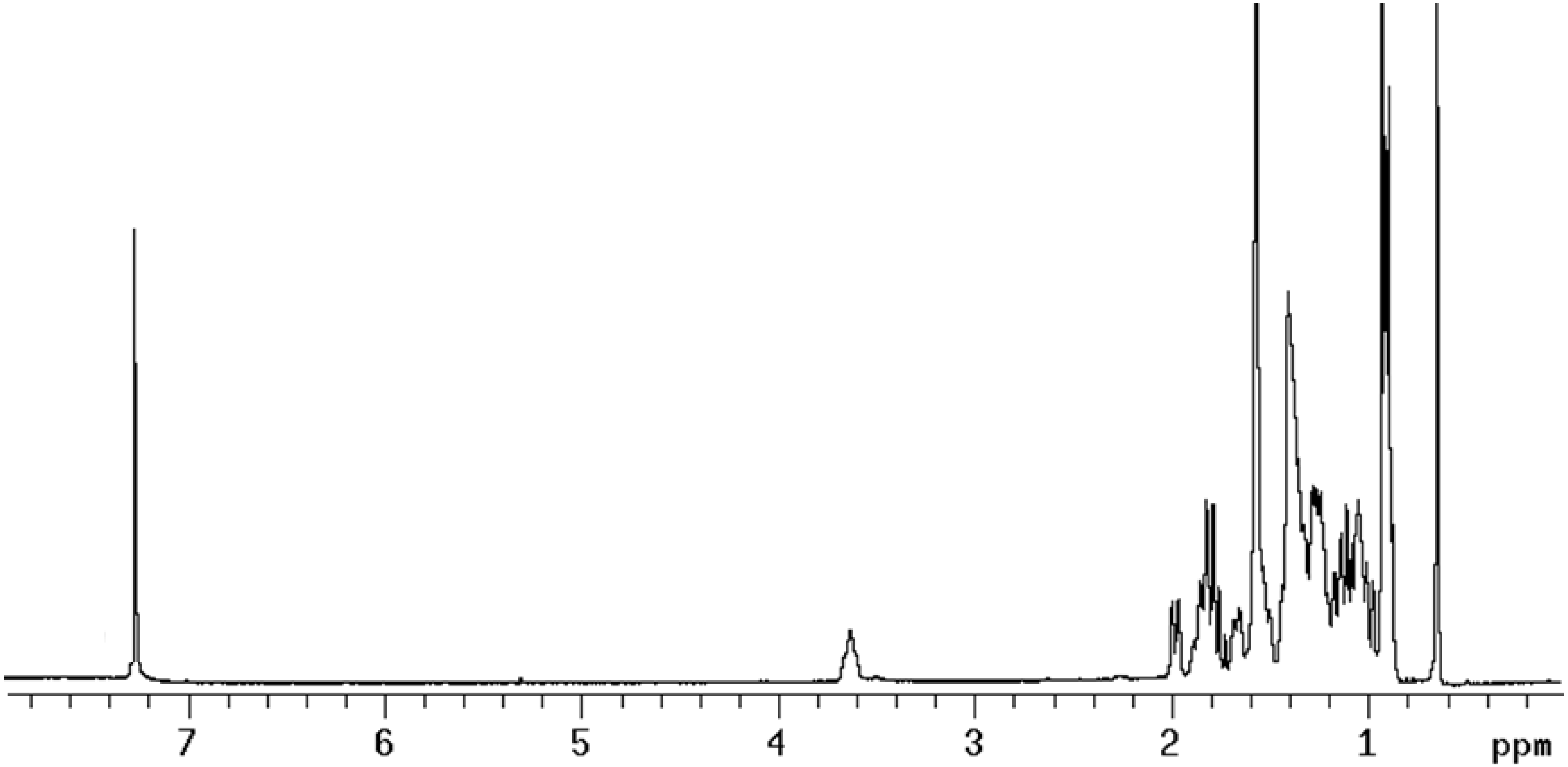

^{1}H NMR (400 MHz, $CDCl_3$) of compound **21**

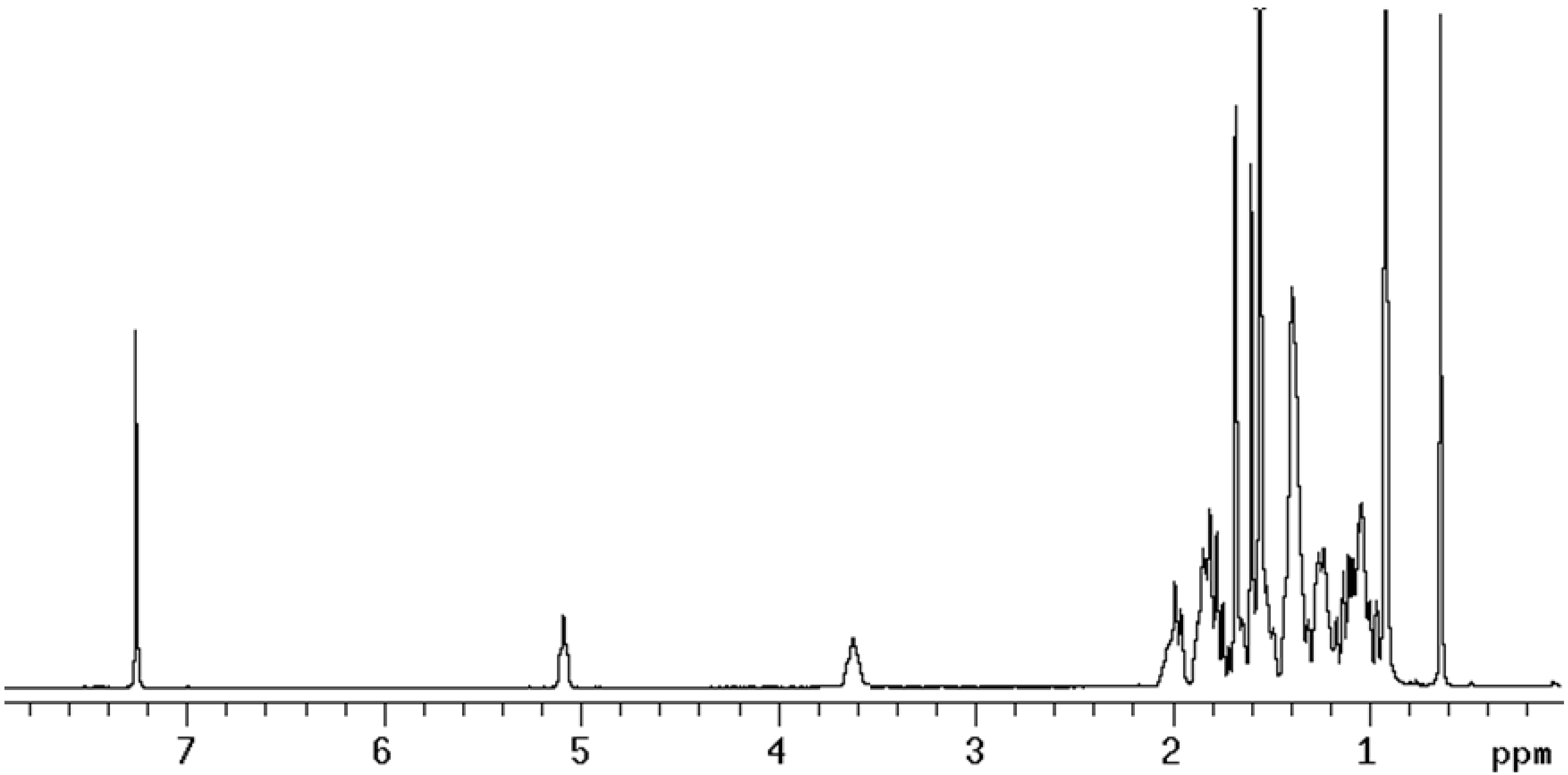

^{1}H NMR (400 MHz, $CDCl_3$) of compound **22**

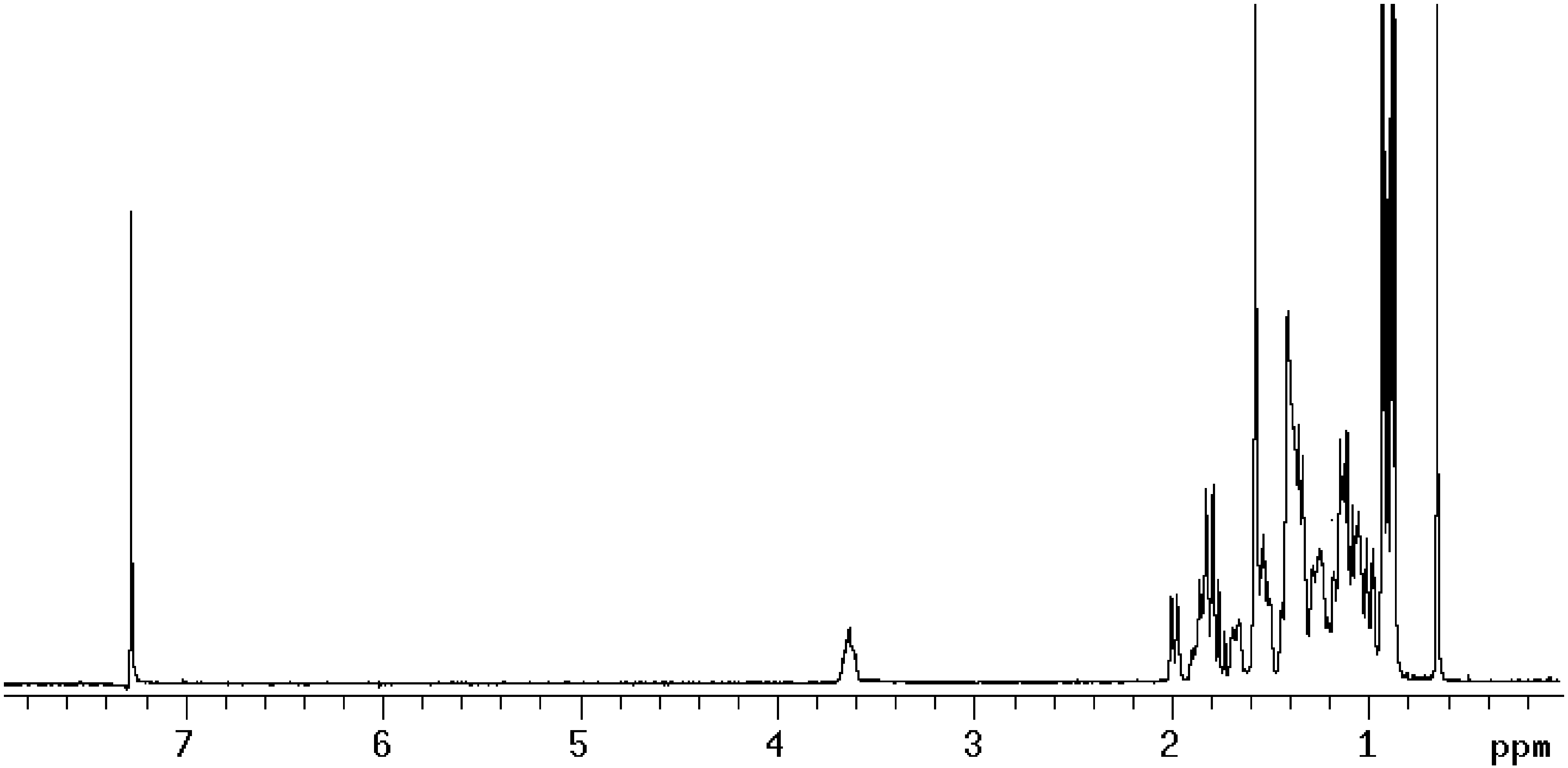

^{1}H NMR (400 MHz, $CDCl_3$) of compound **23**

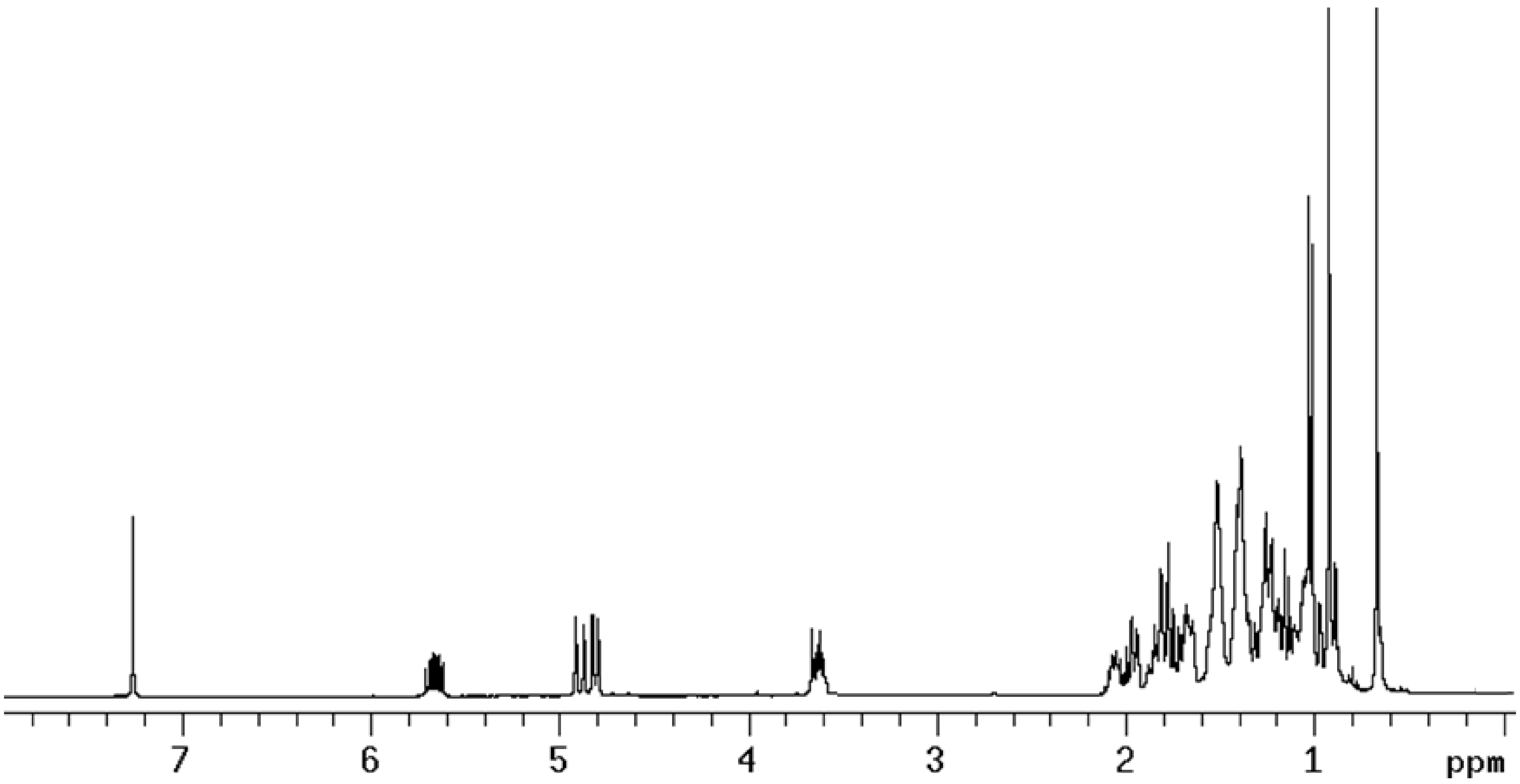

^{1}H NMR (400 MHz, $CDCl_3$) of compound **24**

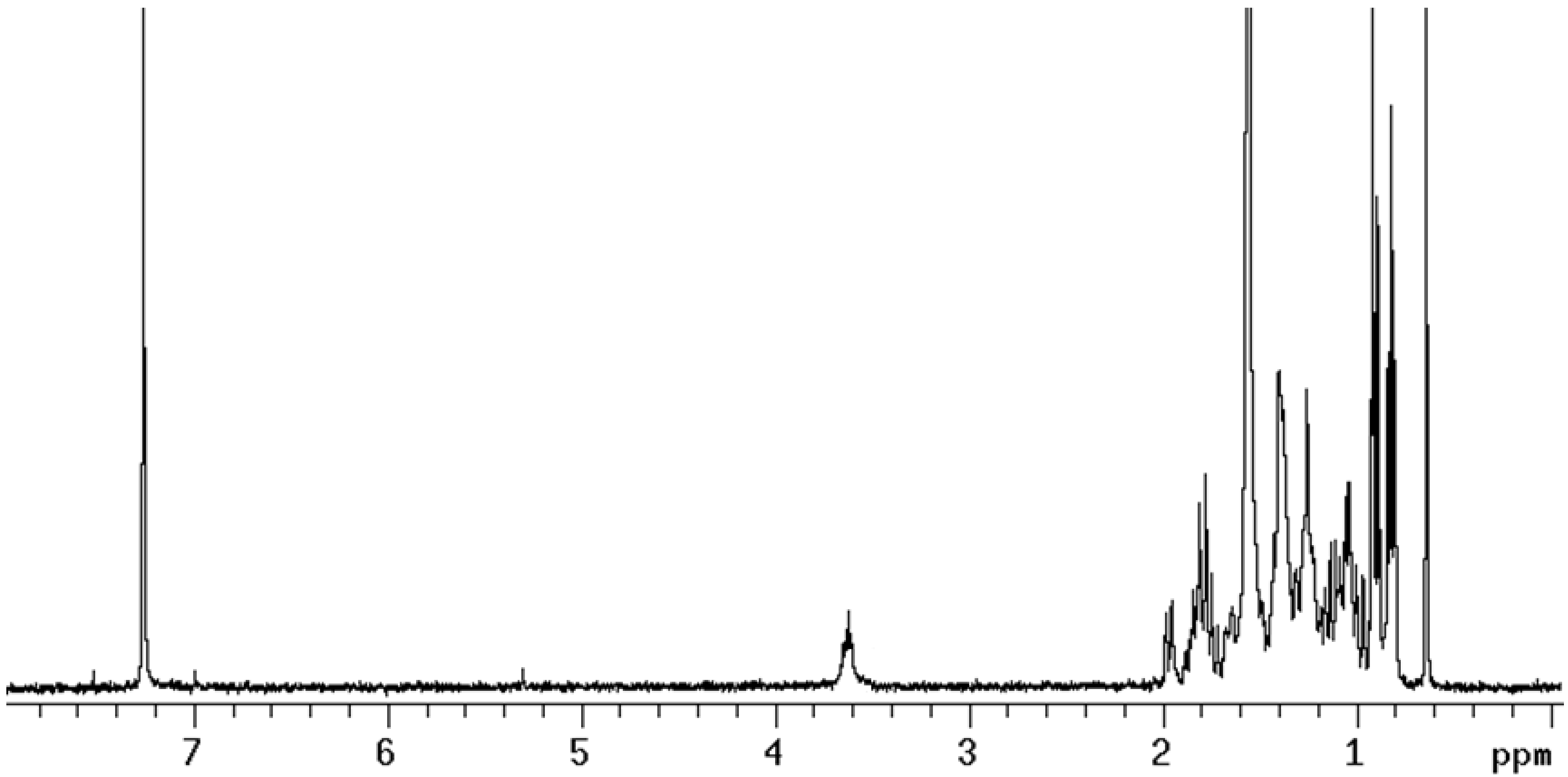

^{1}H NMR (400 MHz, $CDCl_3$) of compound **25**

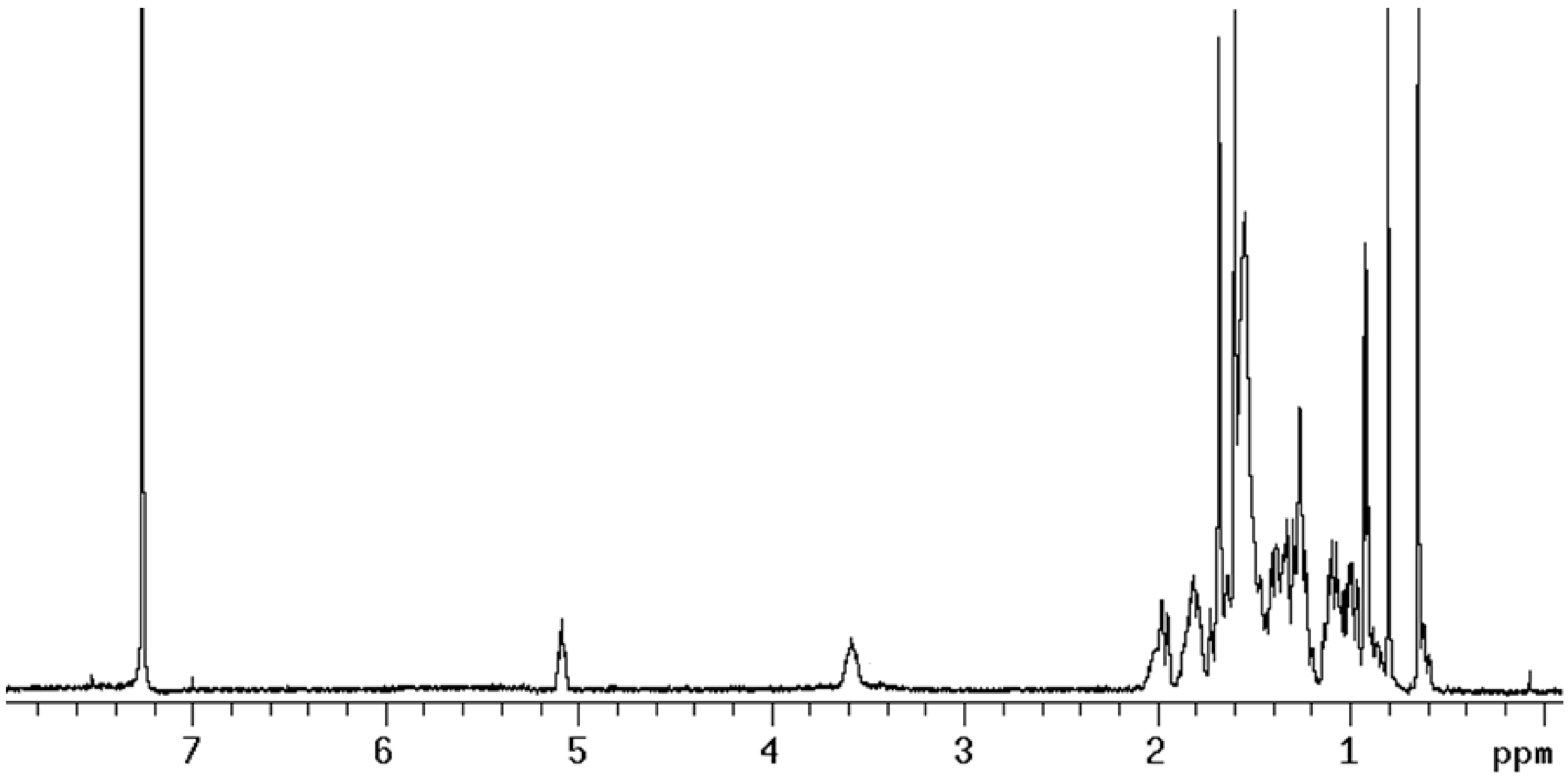

^{1}H NMR (400 MHz, $CDCl_3$) of compound **26**

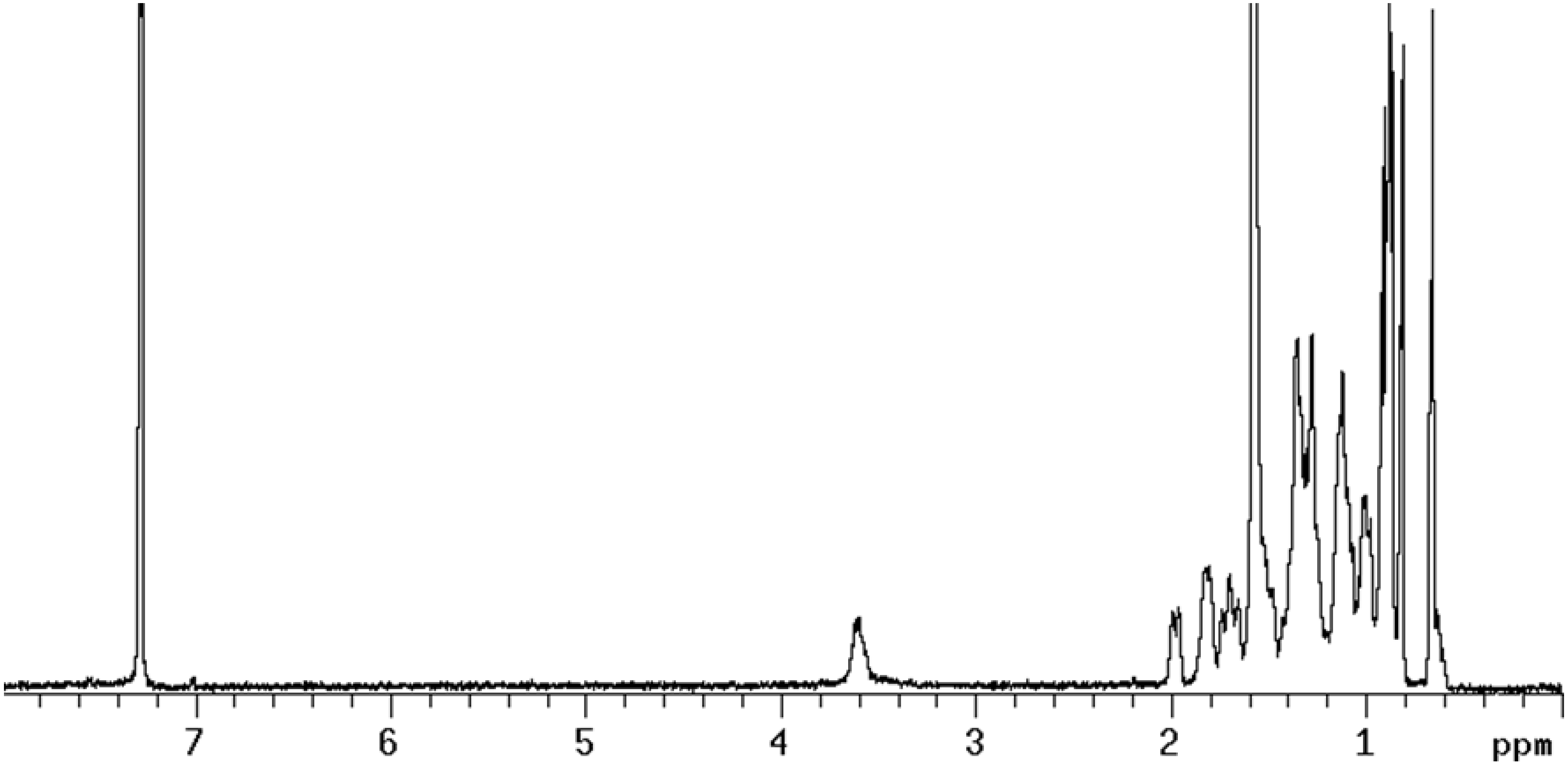

^{1}H NMR (400 MHz, $CDCl_3$) of compound **27**

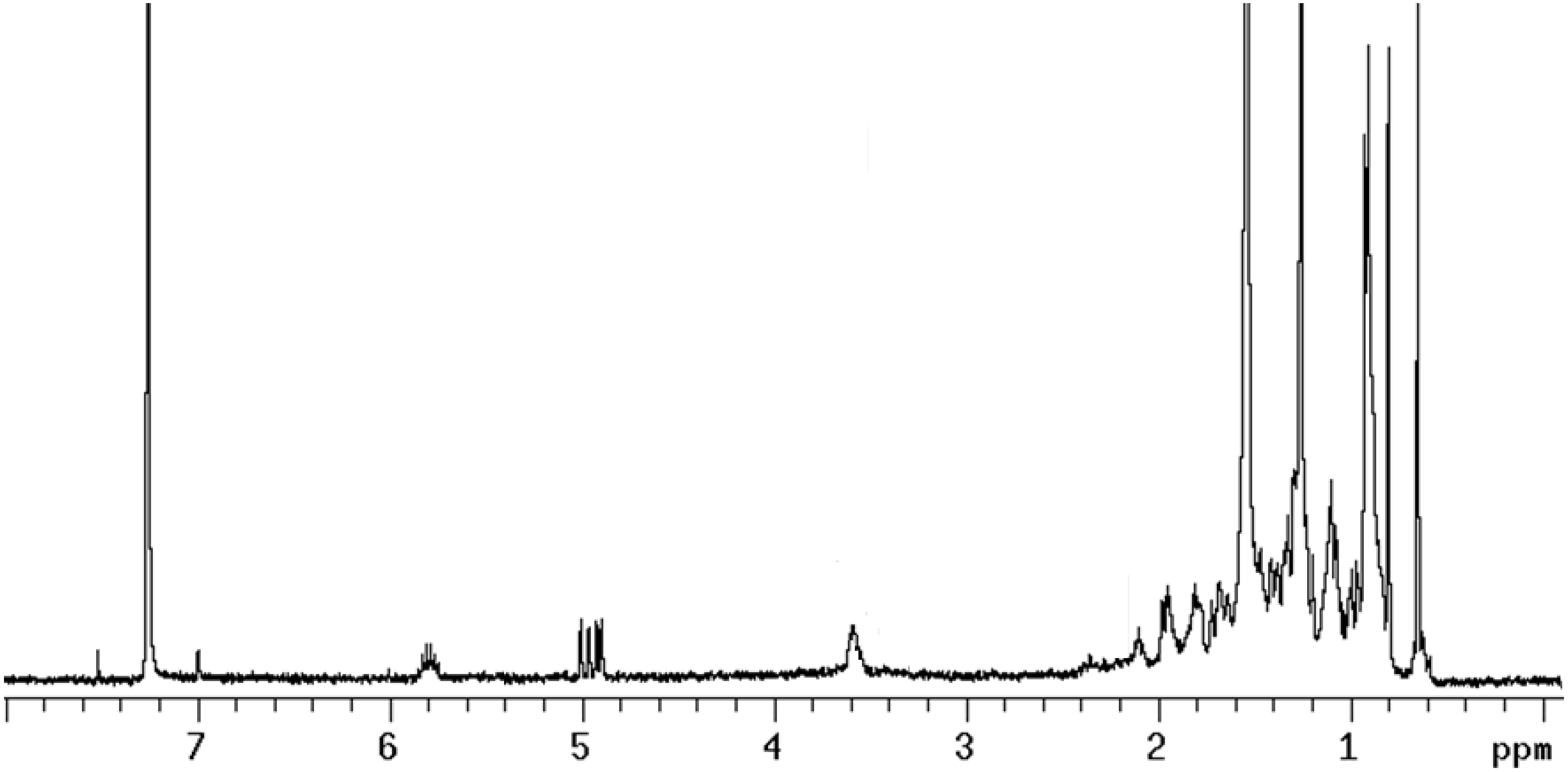

^{1}H NMR (400 MHz, $CDCl_3$) of compound **28**

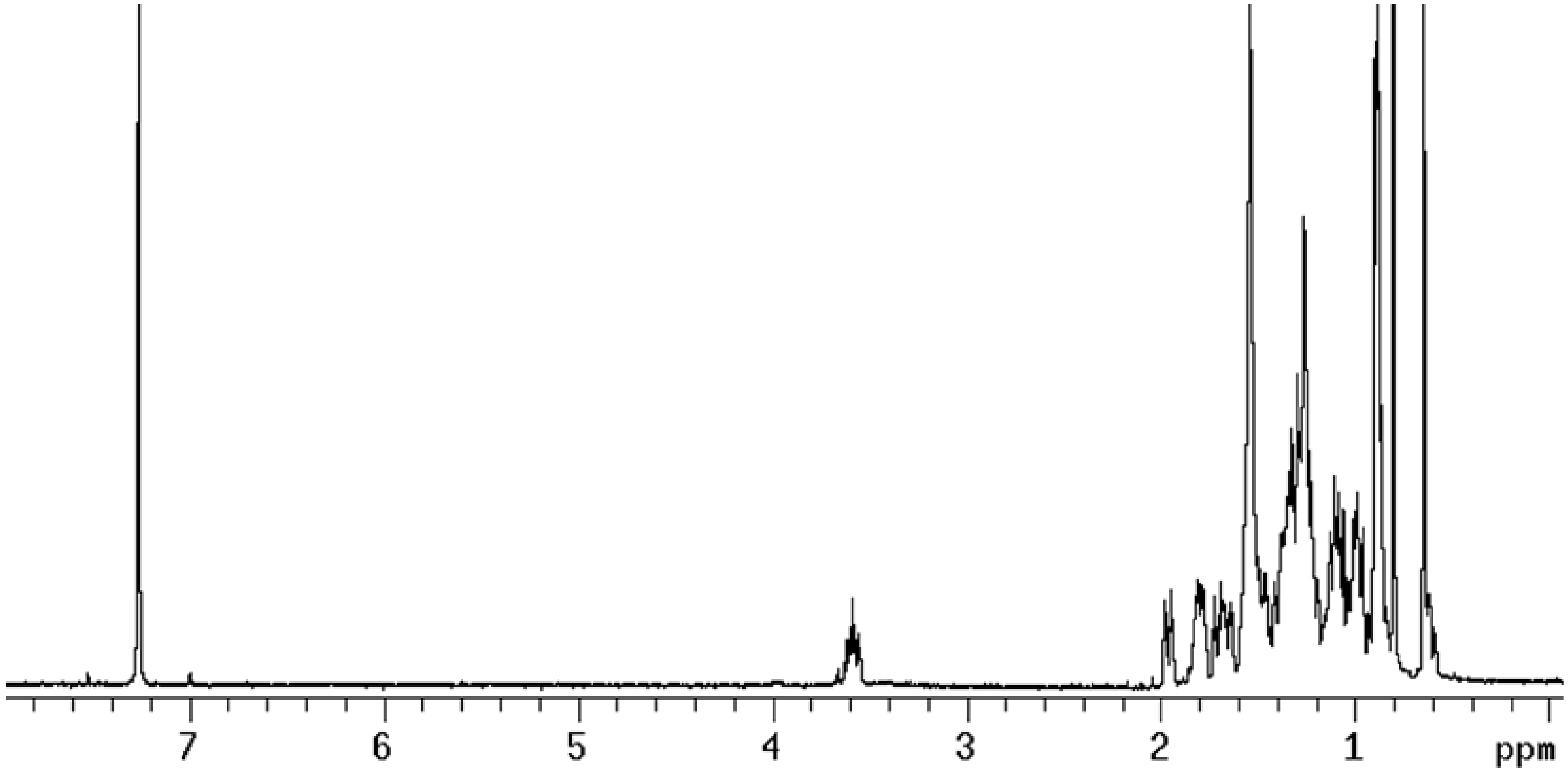

^{1}H NMR (400 MHz, CD_3OD) of compound **29**

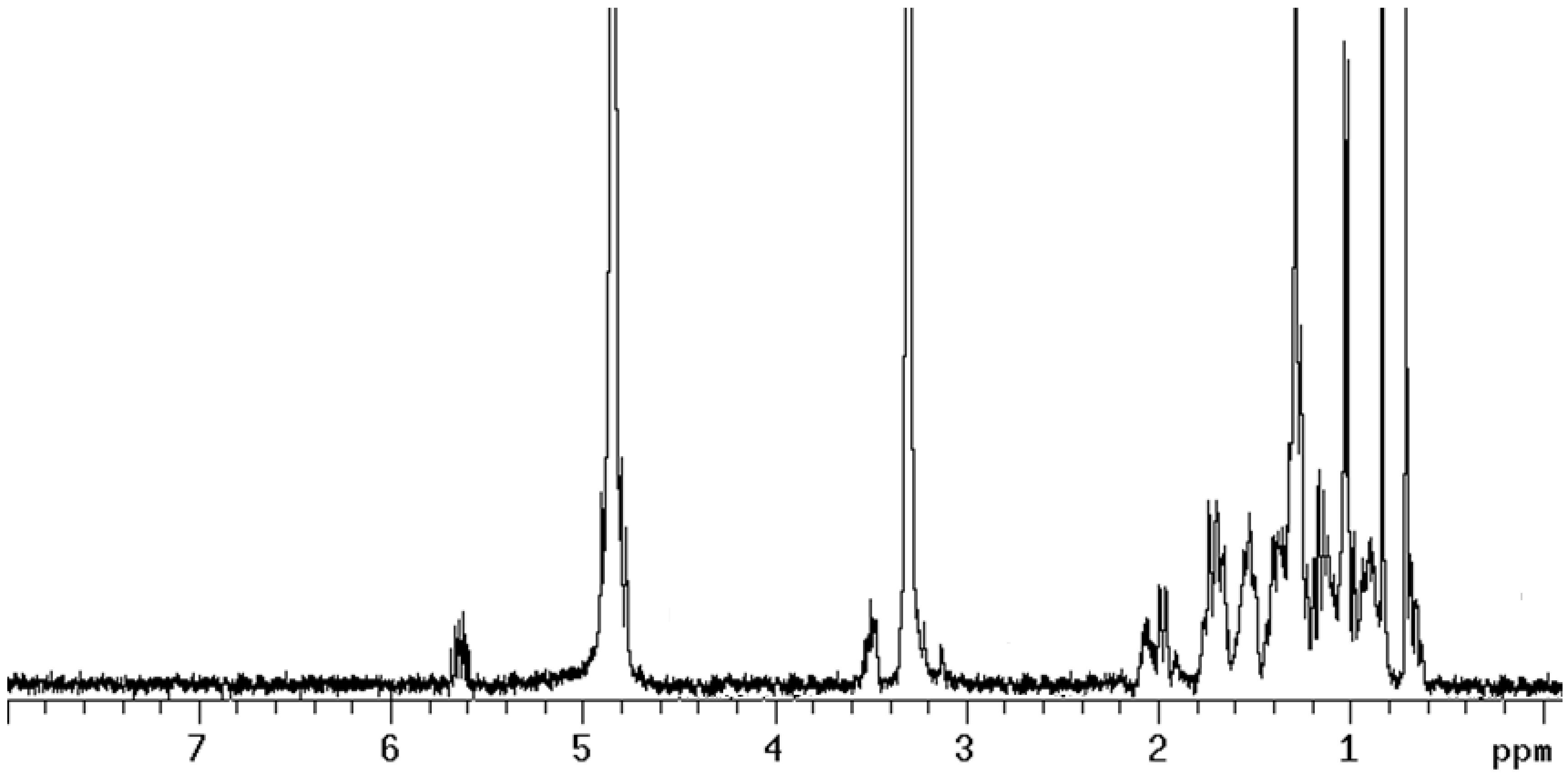

^{1}H NMR (400 MHz, CD_3OD) of compound **30**

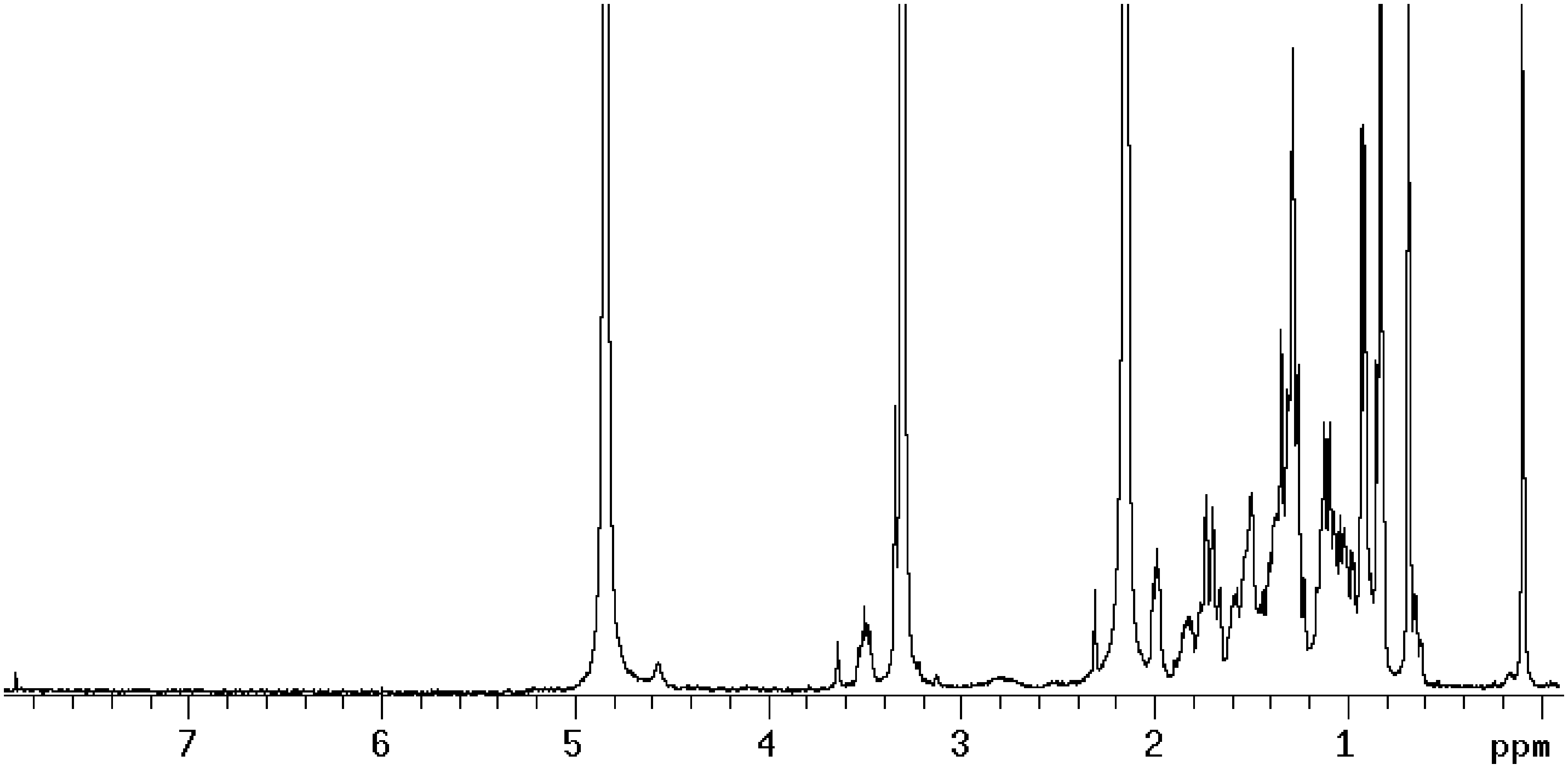

Supplement: Supplementary Information [file srep43290-s1.pdf]
